# Proceedings of the 33rd Congress of the Italian Society of Neonatology, Lombardy Section, 31 January - 1 February 2020: Bergamo, Italy. 31 January-01 February 2020

**DOI:** 10.1186/s13052-020-00934-0

**Published:** 2020-12-29

## S1 Strategies for improving breastfeeding in fragile newborns admitted in NICU: a yearlong experience

### Marco Alessandrini, Paola Coscia , Silvia Cioffi, Lisa Carzaniga, Sabina Paganini, Romina Paganin, Sara Perelli, Roberta Restelli, Laura Ilardi

#### Neonatology and Neonatal Intensive Care Unit - ASST Grande Ospedale Metropolitano Niguarda, Milan, Italy

##### **Correspondence:** Marco Alessandrini (marco.alessandrini@ospedaleniguarda.it)

**Background**

Breastfeeding is a challenge for very preterm and/or sick infants, due to the difficulties related to the underlying pathology and to the specific nutritional requirements. Unfortunately, breastfeeding is too often underestimated when evaluated for neonates health.

Despite proven benefits of breast milk to fragile neonates admitted in the NICUs, its translation and application into best practices, policies, and procedures is still limited; only a small percentage of newborn is discharged with breastfeeding or exclusive human milk feeding. [1-4]

**Materials and methods**

Our NICU has taken charge of this aspect by creating a professionals task force, with nurses, neonatologist and a psychologist, involved and properly trained to support newborns and mothers, practically and emotionally, improving their breastfeeding skills. We have been meeting periodically since October 2018 when our task force was created, then we began collecting data in January. During our meetings we tried to simplify the breastfeeding process, from birth to discharge in a few steps, with the purpose to guide our clinical practice and verify our progresses.

We also created procedures and toolkits to improve mother's empowerment through the whole experience.

**Results**

The increase in NICU-discharged infants with exclusive breastfeeding has not been significant; nevertheless we’ve been able to work on educating and preparing mothers about the importance of human milk, and we doubled our percentage of newborns discharged with exclusive human milk feeding.

**Conclusion**

It is proven that some neonates in NICU, especially those with chronic diseases, will never be able to be exclusively breastfed; nevertheless it is possible to exclusively feed them with human milk by using alternative methods.

A NICU with an improved work environment and better trained nurses and neonatologists can guarantee breastfeeding support to mothers and babies and therefore achieve a higher rate of newborns discharged home with exclusive human milk feeding.

Promoting a culture of breastfeeding in the NICU is feasible; mothers need to be involved and properly prepared for the challenge through education, reinforcing milk expression practices, and facilitating skin to skin contact.

**References**

1. Diane l., et al, Ten steps for promoting and protecting breastfeeding for vulnerable infants, *Journal of Perinatal and Neonatal Nursing*, 2004;18(4):385-396

2. Nyqvist KH, et al, Expansion of the ten steps to successful breastfeeding into neonatal intensive care: expert group recommendations for three guiding principles, J*ournal of Human Lactation*, 2012;28(3):289-296

3. Nyqvist KH, et al, Expansion of the Baby-Friendly Hospital Initiative ten steps to successful breastfeeding into neonatal intensive care: Expert group recommendations, *Journal of Human Lactation*, 2013;29(3):300-309

4. Sunny G, et al, Characteris tics of the NICU work environment associated with breastfeeding support, *Adv Neonatal Care*, 2014: 14(4):290-300

## A1 Osteopath and newborn: our experience

### Andrea Arcusio^1^, Maria Cristina Villa², Filippo G. Porcelli²

#### ¹Dept. Of Rehabilitative Medicine - San Giuseppe Hospital – Multimedica – Milano (Italy); ²Dept. Of Neonatology - San Giuseppe Hospital – Multimedica – Milano (Italy)

##### **Correspondence:** Andrea Arcusio (andrea.arcusio@gmail.com)

**Background**

Osteopathy is a manual conservative diagnostic-therapeutic system classified among complementary medicines. It is a non-invasive and painless discipline that evaluates the musculoskeletal, fascial, visceral and cranio-sacral system. Several reports in the literature indicate that in the neonatal context osteopathy allows to solve somatic dysfunctions that originate from positions held too long in the uterus and/or from perturbations of delivery kinetics; it also allows to prevent the onset of postural and organic-functional disorders, creating the mechanical prerequisites for a regular growth and an harmonic and physiological psychoneuromotor development. Osteopathic treatment involves one or more sessions, depending on the symptomatology and on alteration of tissue plasticity found in the manual evaluation: in particular it is directed to the tissues found to be relatively less plastic and linked in an anatomic-functional sense with the symptoms that the newborn manifests. The caregivers' compliance is fundamental for obtaining good clinical results: parents are then trained in the management of daily care according to the basic neuromotor patterns, for the promotion of mutual skills (in accordance with Brazelton’s theories).

**Materials and methods**

In our Department of Neonatology from May 2018 to November 2019 were born 2186 children, 309 of which, depending on the anamnestic risk factors and/or clinical objectivity, underwent osteopathic treatment in the ward (see Table 1).

**Results**

45 newborns needed only one treatment in the ward, while 264 needed to continue the outpatient treatment: of these, 142 completed the proposed course, with a positive clinical result in 100% of cases. The first treatment was performed in the ward within the first 72 hours of life, while the following sessions were performed as outpatients with the active involvement of caregivers. The more frequently founded symptoms and their correlations with the risk factors are reported in Table 2:

Even with the gestational age limits and the limited numbers of patients, our data suggest that 60% of treated babies were born from dystocic delivery, and that 25% of them presented mainly an alteration of the rotational side of the head. 33% of the children were treated because of abnormalities at clinical examination; of these, an alteration of the rotational side of the head was found in 11% of cases and a postural alteration of the foot in 8% of cases.

**Conclusions**

Osteopathic counseling has proved useful in the care of newborns with risk factors.

**Table 1 (abstract A1).** Population examined

RISK FACTORS	DESCRIPTION	NUMBER OF TREATED NEONATES
Twins		6
Prematurity	G.A. < 37 weeks	17
Perinatal asphyxia/depression	APGAR Score < 7 – funicolar pH < 7.1 – emergency cesarean delivery	28
SGA – LGA neonates	Weight < 3° c.le or > 90° c.le	47
Dystocia	Vacuum extraction, precipitous delivery, protracted labor, abnormal presentation, funicular tours	137
Abnormalities at Clinical examination (ACE)	Skeletal-axial and/or appendicular postural alterations	74

**Table 2 (abstract A1).** Correlation between symptoms and risk factors

SYMPTOMS	% OF CASES	DUE MAINLY TO	NUMBER OF TREATMENTS (AVERAGE)
Cranio- facial asymmetry and related functional alterations	57	• Dystocia • SGA – LGA • ACE	5 5 4
Changes in the postural configuration of the pelvis and/or lower limbs	36	• Dystocia • SGA – LGA • ACE	4 2 3
Gastro-colic functional alteration	31	• Dystocia • SGA – LGA • ACE	5 4 4
Alterations of Column, Upper Limbs and/or Lower Limbs Tone	15	• Dystocia • Perinatal asphyxia/depression	3 2

P.S.: In many cases more symptoms and/or conditions were simultaneously present in the same child

## A2 Unexpected respiratory distress in the delivery room: a case of tracheal atresia

### Elvira Bonanno, Antonietta Distilo, Gabriella Nigro, Francesco Morrone, Mara Salvia, Gianfranco Scarpelli

#### Department of Neonatology, Annunziata Hospital, Cosenza, Italy

##### **Correspondence:** Elvira Bonanno (elvirabonanno@libero.it)

**Background**

Tracheal atresia (TA) is an uncommon congenital malformation (1:50.000/100.000) with a male predominance and a high mortality rate. The defect may be isolated or occur in association with other congenital abnormalities.

**Case report**

We describe a small gestational 34 week male (46 XY-caryotype) newborn by cesarean section. On sonography, polyhydramnios was noted. At delivery, the infant showed absence of cry and independent breath, diffuse cyanoses, a hypo-expanded thorax. Ventilation was applied via a balloon mask because of respiratory distress and cyanosis, but a sufficient response could not be obtained. Endotracheal intubation was attempted, but was unsuccessful because the endotracheal tube could not be advanced below the level of the vocal cords. Chest compression were initiated. An umbilical vein catheter was place. An attempt was made to intubate the esophagus (assuming that if there were a tracheoesophageal fistula). The lungs could not be ventilated. Resuscitative efforts were terminated 25 minutes after delivery. A post-mortem examination was performed with parental consensus and revealed diffuse cyanotic status, a tract of 3 cm of trachea distantly ending in a blind pouch and without tracheoesophageal fistulae and enlarged bulky lungs connected to each other by a common thin-walled bronchus were documented. A diagnosis of tracheal atresia was made. No other congenital malformations were detected, and a normal vascularized umbilical cord was observed. Histological examination showed a normal conformed larynx and scratchily cartilaginous disks only in the proximal tract of the short trachea.

**Conclusion**

Tracheal atresia is a severe congenital disorder with often an unexpected emergency presentation. Respiratory distress at birth with absence of audible cry, cyanosis and the impossibility of tracheal intubation are the main clinical presentations. Prenatal diagnosis of TA is possible through antenatal ultrasonography that may show enlargement and hyperechogenicity in the lungs, enlargement of the upper airways, hydrops fetalis, and polyhydramniosis. Prenatal diagnosis of TA is confirmed by fetal MRI. Although tracheal atresia is usually lethal, it has recently become possible to bypass the airway obstruction and establish adequate ventilation by the EXIT procedure (Ex Utero Intrapartum Treatment). Several successful cases have been reported. Accurate antenatal diagnosis is essential for a patient with TA to have a chance for successful EXIT application.

Informed consent to publish has been obtained from the parents.

## A3 An atypical neonatal respiratory distress

### Francesca Cortinovis^1^, Manuela Condò^2^, Carla Maccioni^2^, Emilia Massironi^1^, Francesco Morandi^1^

#### ^1^ ASST Lecco, UOC Pediatria- Neonatologia, Ospedale “L. Mandic” Merate (Lc), Italy; ^2^ASST Lecco, Terapia Intensiva Neonatale e Pediatria, Ospedale “A. Manzoni”, Lecco, Italy

##### **Correspondence:** Francesca Cortinovis (f.cortinovis@asst-lecco.it)

**Background**

We report an interesting case of a neonatal respiratory distress due to rib cage deformities caused by skeletal dysplasia.

**Case Report**

N.F. was born at 37+3 weeks g.a. by caesarean section due to IUGR, severe polyhydramnios and breech presentation, by consanguineous parents of sub-Saharan origin. During pregnancy invasive prenatal diagnosis was performed with Karyotype and CGH Array analysis, both normal. At birth the weight was 2380 g (8°p.le), the length 47,5 cm (34 p.le), CC 33,6 cm (56° p.le). In the first hours she developed progressive respiratory distress and was transferred to NICU where was assisted with n-CPAP for 22 days (FiO2 max 0,3), followed by HFNC for other 15 days. Chest X-Rays showed lungs hypoexpansion and dysmorphic ribs with increased radiotransparency, compatible with prenatal ribs fractures (see Fig.1, rib cage deformities). Little thoracic expansion was also clinically evident. Imaging was completed with long bone x-rays that showed fractures in proximal and distal right femoral epiphysis and distal left femoral epiphysis. No vertebral fractures neither cranial fractures were detected. Echocardiography was normal as well as cerebral and abdomen ultrasound scan. Considering this clinical presentation osteogenesis imperfecta was suspected and genetic analysis by NGS was performed. However, no abnormal variants in the analysed genes were detected. LEPRE gene analysis is still ongoing. Calcium and phosphate blood levels were normal, as well as PTH, with little elevation of alkaline phosphatase. Accordingly with Reference Centre, the baby underwent two course of bisphosphonate therapy (neridronate) and began cholecalciferol therapy at initial dosage of 1000 UI per day, reduced to 500 UI after elevation of 25OHvitaminD. The negativity of genetic analysis and the hormonal assays suggest that the clinical presentation of the baby could be due to severe prenatal maternal vitamin D deficiency. Mother blood 25OHvitamin D was low and was therefore supplemented. However, phenotypical features and suboptimal growth in the following months could be due to another genetic condition that has to be investigated.

**Conclusion**

Osteogenesis imperfecta (OI) is a heterogeneous group of connective tissue syndromes characterized primarily by liability to fractures associated with other features e.g. blue sclerae, dentinogenesis imperfecta, secondary deformations. Many genes are currently known and associated with different forms ranging from perinatal lethal to mild forms.

Prenatal fractures can be due to severe forms of OI but differential diagnosis must be done with other condition e.g. storage diseases, severe rickets, other skeletal dysplasias.

Informed consent to publish has been obtained from the parents.

**Fig. 1 (abstract A3).**
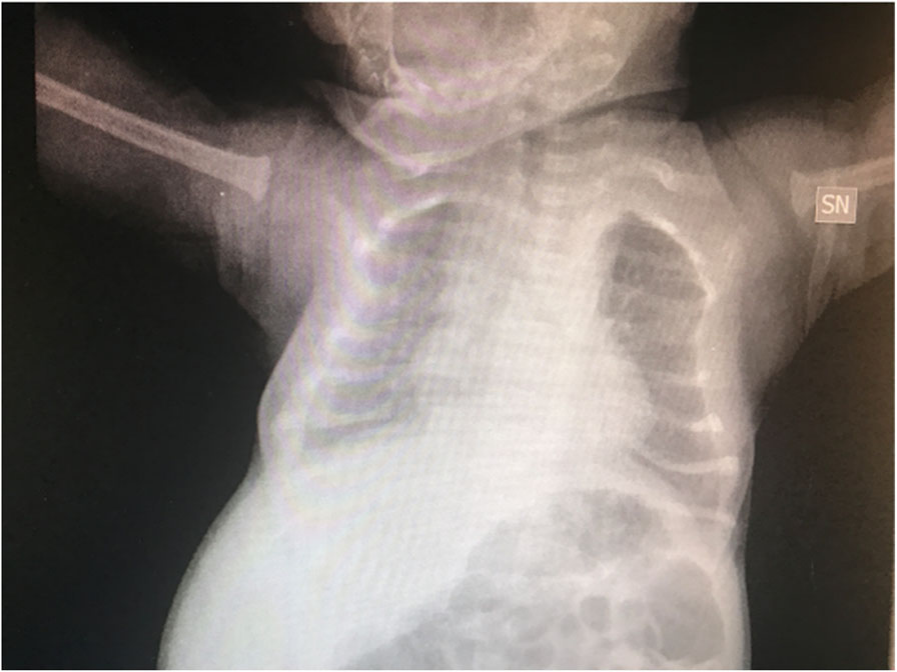
Rib cage deformity

## A4 An unusual case of butalbital-induced neonatal withdrawl syndrome combined with caffeine-induced intra-uterine growth restriction: a clinical and relational challenge for neonatologist

### Maria Elena Capra^1^, Nicoletta De Paulis^1^, Giulia Vezzoni^3^, Marco Cirronis^2^, Giacomo Biasucci^1^

#### ^1^UOC Pediatrics and Neonatology, G da Saliceto City Hospital, Piacenza, Italy; ^2^Pavia Poison Control Center- National Toxicology Information Center – Maugeri Clinical and Scientific Institute IRCCS and University of Pavia, Pavia, Italy; ^3^ Fellowship Programme of emergency Medicine, University of Pavia,Pavia, Italy

##### **Correspondence:** Nicoletta De Paulis (depaulisnicoletta@yahoo.it)

**Background**

Substance use among pregnant women is a major public health issue. Abuse of drugs and opioid has increased dramatically in the last years. Prolonged in-utero drug exposure may result in neonatal withdrawal syndrome (WS) , which is an emerging problem in NICU worldwide. Butalbital is an intermediate acting barbiturate with a good oral bioavailability. It acts as central nervous system depressant. It is often commercialized in combination with other analgesic drugs of headache treatment. Butalbital should not be used in pregnancy and during lactation because it crosses the placenta barrier and it passes through breastmilk. As most barbiturates, its overuse can be linked to dependence and WS. WS can start 8 to 36 hours after the last drug assumption, it lasts from 2 to 15 days and may be lethal. In literature very few cases have been described in neonates born from mothers who use butalbital, propifenazone and caffeine during pregnancy. Treatment of butalbital’s WS is based on phenobarbital and or benzodiazepines. A tactful yet early and direct parents’ counseling is of pivotal role in the early identification of families at risk for substance abuse.

**Case report**

A male baby boy was born at 37+4 weeks by cesarean delivery due to maternal cholestasis and intra-uterine growth restriction (IUGR); birth weight was 2120 gr (2° centile). No drugs assumption was reported during pregnancy. At birth he showed normal adaptation to extrauterine life but a persistent, isolated tachycardia (FC 185 bpm) and jitteriness. He was conducted in NICU. Blood exams for glucose, electrolites, PCR and electrocardiogram at two hours were normal. Nevertheless, Neonatologist were not persuaded and made a one-to-one counseling with his father on the baby’s conditions. The father agreed to report it to the mother (still under surgical treatment). After this counseling, the father told doctors that he had found at home a lot of empty packages of suppository composed by butalbital 150 mg, caffeine 75 mg and propifenazone 375 mg. On the hypothesis of neonatal abstinence syndrome, urine toxicological exams were perfomed and high phenobarbital levels were found. Finnegan Score’s evaluation was performed and, after several high scores, phenobarbital therapy was started. Therapy was reduced and stopped after some days of good neurological conditions. The family was entrusted to the Social Service of our city.

**Conclusions**

This case report is very interesting as the drug we reported is an old-fashioned one, rarely used nowadays. Having no positive anamnesis for drug assumption and/or abuse, Neonatologists were not previously alarmed and considered withdrawal syndrome not at first instance. Assumption of caffeine higher than 300 mg daily in pregnancy is linked to IUGR, whereas assumption of butalbital is linked to withdrawal syndrome. In our case, we believe that both these effects were combined, making the case even more tricky and interesting.

Informed consent to publish has been obtained from this patient

## A5 Respiratory syncytial virus bronchiolitis and supraventricular tachycardia in neonatal period of life

### Daniela Doni, Luisa Impagnatiello, Silvia Barzaghi, Maria Luisa Ventura

#### Neonatal Intensive Care Unit, MBBM Foundation, San Gerardo Hospital, Monza, Italy

##### **Correspondence:** Daniela Doni (danieladoni@libero.it)

**Background**

Respiratory syncytial virus (RSV) is the most common cause of lower respiratory tract infections in infants. Supraventricular tachycardia (SVT) is one of the most important cardiovascular manifestations of RSV. The virus can be detected in myocardial tissue and through invasive, inflammatory or toxic effects causes acute or chronic rhythm disturbances. Unusual SVT have been reported during RSV infection in patients with structurally normal hearts. Usually it doesn’t recur after the acute event. No association with β-agonist therapy or hypoxia. In patients with bronchiolitis by RSV and structurally normal hearts, SVT usually responded to medication without the need for cardioversion and it is self-limited not requiring prolonged therapy.

**Case report**

Infant born at 39+3 weeks of gestational age by spontaneous delivery after a normal pregnancy, appropriate for gestational age. At 28 days of life he was hospitalized for respiratory symptoms. High flow nasal cannula (HFNC) was started. Nasopharyngeal aspirate positive for RSV. We assisted to a gradual improvement of mechanical respiratory until HFNC suspension after five days. He didn’t need β-agonist or steroid therapy. After two days, sudden onset of tachycardia with HR > 200 bpm. The child was responsive with pale skin, capillary refilling of 3-4 seconds, cold extremity. O2 saturation was 100% in room air, BP 95/55 mmHg. An electrocardiogram (ECG) was performed (Figure 1). Diving first was performed and then two bolus of adenosine (0,1 and 0,3 mg/kg) with the recovery of sinusal rhythm, HR 176 bpm, without pre-excitation of the ventricle. Forty minutes after, new episode of SVT. A third bolus of adenosine (0,3 mg/kg) was administered with a new recovery and maintenance of regular rhythm. The day after the ECG showed normal PR and RR intervals, normal QTc, no evidence of any accessory pathways. At echocardiography: no evidence of congenital heart disease, physiological PFO. The diagnosis was SVT probably due to an hidden accessory pathways in a structurally normal heart. The patient was monitored with Holter ECG and began therapy with flecainide 3 mg/kg/die twice a day and propranolol 1 mg/kg/die three times a day. No recurrences in the following 48 hours. He was discharge with a cardiac evaluation a month later.

**Conclusion**

All neonates or infants with RSV infection should be HR monitored for detect SVT.

Informed consent to publish has been obtained from this patient

**Fig. 1 (abstract A5).**
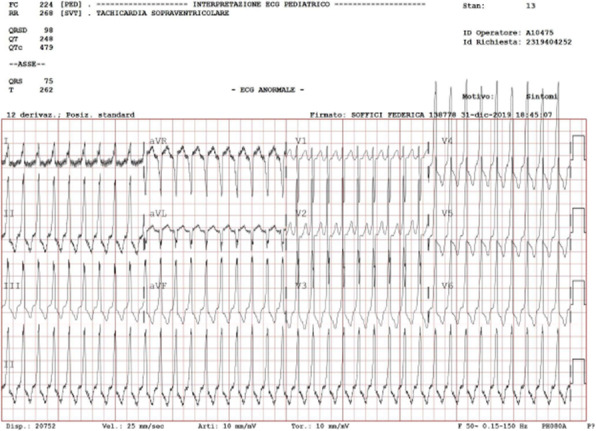
ECG in SVT: FC 224 bpm conducted with aberrancy probably due to accessory pathways, absence of P waves, narrow QRS like a paroxysmal SVT

## A6 Umbilical venous catheter and ectopic atrial tachycardia: a case report

### Elisa Dusi, Maddalena Gibelli, Maria Lorena Ruzza, Sabrina Argirò, Stefano Rizzi, Alberto Podestà

#### ASST Santi Paolo e Carlo, Ospedale San Carlo Borromeo, Milan, Italy

##### **Correspondence:** Elisa Dusi (elisa.dusi@asst-santipaolocarlo.it)

**Background**

The malposition of umbilical venous catheter (UVC) can cause complications such as thrombosis, infection extravasation or tachyarrhytmias. Ectopic atrial tachycardia (EAT) is a relatively common type of supraventricular tachycardia in the pediatric population, and it can be resistant to antiarrhythmic drugs and lead to tachycardia-induced cardiomyopathy. [1-3] We present a case of EAT in a newborn with UVC.

**Case Report**

A 2470 g , 33-week gestation female was born following a pregnancy complicated by assessment of foetal tachycardia during routine control. Delivery was via emergency lower segment caesarean section. Apgar score at birth were 9 (1 minute), 10 (minute), no need of initial resuscitation. On admission the infant had a sinus rhythm with a normal heart rate but on physical examination slight groaning and subcostal retraction were found. UVC was inserted. Directly after introduction of UVC the infant developed a mild tachycardia (heart rate up to 190 beats/min). An anterior-posterior chest X-ray showed the catheter tip onto the right atrium. The UVC was pulled back for 1,5 cm according to radiological findings but mild tachycardia persisted. Her subsequent management included empiric antibiotics administration for a potential neonatal infection During the second day of life we observed complete resolution of respiratory distress but persistence of period of mild tachycardia (170-190 beats/min). An electrocardiogram was diagnostic for EAT (fig.1). Bed-side ecocardiography was performed and showed the tip of UVC in fossa ovale. The UVC was pulled back 2 cm with normalization of the heart rhythm (fig.2) However, the next day EAT reoccurred and we started a propranolol therapy on indication of pediatric cardiologist without success of converting to sinus rhythm. For this reason we referred the infant to our neonatal hub for care.

**Conclusion**

The infant was probably affected by EAT already present during foetal life. In our report the malposition of UVC may play a role as trigger of tachyarrhytmias. Some studies suggest that UVC could cause mechanical distortion of the atria predisposing to development of tachycardia and we observed temporary reversion to normal heart rate after catheter tip withdrawl. This report highlights the need of determining catheter position by chest X-ray or echocardiography to avoid possible complications. This case is also a reminder that UVC can migrate and consideration should be given to serial catheter imaging to reduce catheter- related complications.

Informed consent to publish has been obtained from this patient

**References**

1. Amer A, Broadbent RS, Edmonds L, Wheeler J. Central venous vatheter- related tachycardia in the newborn: case report and literature review. Case Rep Med 2016 6206358

2. Dubbink-Verheij GH,Visser R,Tan RNGB,Roest AAW,Lopriore E, Te Pas AB. Inadvertent Migration of Umbilical Venous Catheters Often Leads to Malposition. Neonatology 2019;115: 205-210

3. Verheij G, Smits-Wintjens V, Rozendaal L, Blom N, Walther F, Lopriore E. Cardiac arrhythmias associated with umbilical venous catheterisation in neonates. Case Reports 2009; 2009: brc 20091778

**Fig. 1 (abstract A6).**
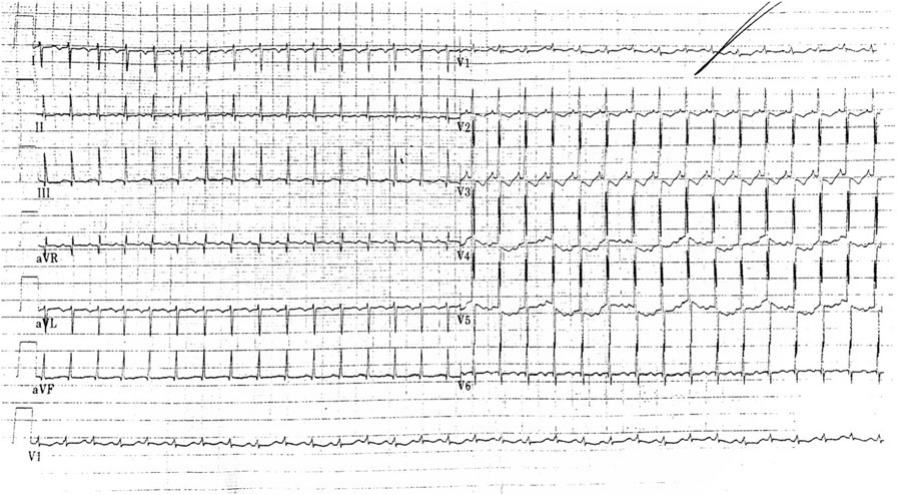
Electrocardiogram shows EAT

**Fig. 2 (abstract A6).**
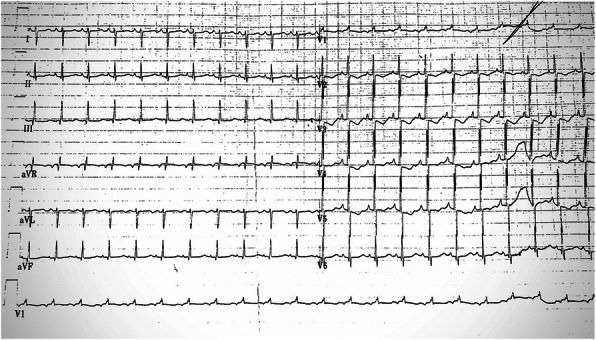
Electrocardiogram shows normal heart rhythm

## A7 An extreme case of hypertrophic cardiomyopathy in in a child of poorly controlled insulin dependent gestational diabetes

### Chiara Gertosio^1^, Mariasole Magistrali^1^, Lucia Schena^1^, Rosario Ippolito^1^, Enrico Tondina^1^, Claudia A Codazzi^2^, Rosa M Cerbo^3^

#### ^**1**^Neonatal Intensive Care Unit, Fondazione IRCCS Policlinico San Matteo, University of Pavia, Italy; ^**2**^ Pediatric Cardiology, Department of Pediatric, IRCCS San Matteo Hospital Foundation, Pavia, Italy; ^**3**^ Neonatal Intensive Care Unit, Fondazione IRCCS Policlinico San Matteo, Pavia, Italy.

##### **Correspondence:** Enrico Tondina (e.tondina@gmail.com)

Gertosio C., Magistrali M. and Schena L. contributed equally to the writing of the work

**Background**

Moderate or severe gestational diabetes (GD) increases the risk of foetal and neonatal complications. Hypertrophic cardiomyopathy is one of the main complications. We report a case of severe hypertrophic cardiomyopathy in a child of poorly controlled insulin dependent GD in pregestational maternal diabete survived post-natal care.

**Case report**

E.A. was born at 37 weeks + 4 days, by caesarean section for cardiotocographic tracing alteration, birth weight 3840 gr (large for gestational age). Severe hypertrophic cardiomyopathy (CHD) was found in fetal echocardiography, for this reason the mother was informed about the possibility of perinatal death and NICU was warned. Actually at birth the patient needed resuscitation and she was prontly admitted to NICU. The echocardiographic evaluation (figure 1), performed at birth and in the first days of life, confirmed severe CHD (septum thickness 12,7 mm at birth,), affecting especially the left ventricle with signs of obstruction of left efflux, without obstruction of efflux in the pulmonary district. Furthermore, patent Botallo duct was detected with a bidirectional shunt. In consideration of the hight risk of low cardiac output syndrome, prostaglandin and beta-blocker therapy (esmolol continuously i.v. for the first three days) were started early at birth.The child presented no other complications due to maternal diabetes, except for macrosomia. Considering the severity of the CHD, to rule out other causes of hypertrophy, genetic investigations (next generation sequency ongoing reporting), skin and muscle biopsy (in order to investigate mitochondrial respiratory chain, with negative results) were performed. The instrumental and laboratory cardiac monitoring during the hospitalization revealed a gradual improvement in cardiac hemodynamics, unless the persistence septum thickness (highest measure 16 mm). The cardiological follow-up after the discharge confirmed the progressive improvement of the clinical condition (thickness reduction of all walls expecially septum thickness 9,7 mm), validating the hypotesis of CHD gestational diabete-related.

**Conclusion**

Severe GD increases the risk of foetal and neonatal complications. [1] Numerous epidemiological studies have demonstrated a strong correlation between GD and significantly elevated risk of CHD in the offspring of affected mothers. [2,3] Precise risk of hypertrophic cardiomyopathy is not known, but severe clinical form is exceptional. Our case report shows that despite a poor outcome at prenatal diagnosis, prompt neonatal care and close follow up allow survival at birth, regular growth and normal psychomotor development at least in the first months of life. We do not know if the regression of cardiomyopathy will be complete, investigations for genetic diseases are still ongoing, but it is important to point out that a correct prenatal diagnosis and adequate post-natal treatment ensure the best chance of survival.

Informed consent to publish has been obtained from this patient

**References:**

1. Mitanchez D. Fetal and neonatal complications of gestational diabetes: perinatal mortality, congenital malformations, macrosomia, shoulder dystocia, birth injuries, neonatal outcomes. J Gynecol Obstet Biol Reprod (Paris). 2010 Dec;39(8 Suppl 2):S189-99.

2. Basu M. Garg V Maternal hyperglycemia and fetal cardiac development: Clinical impact and underlying mechanisms. Birth Defect Res. 2018 Dec 1;110(20):1504-1516.

3. Zabihi S, Loeken MR. Understanding diabetic teratogenesis: where are we now and where are we going? Birth Defects Res A Clin Mol Teratol. 2010 Oct; 88(10):779-90.

**Fig. 1 (abstract A7).**
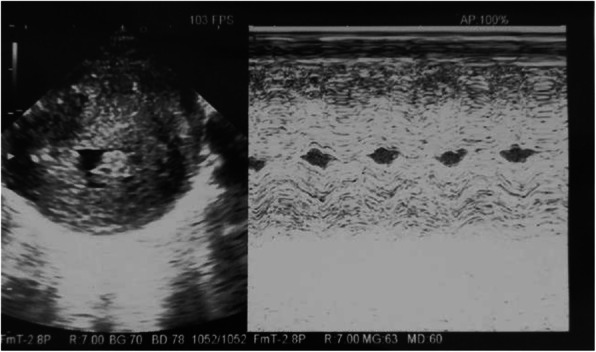
Severe hypertrophic cardiomyopathy

## A8 Polyhydramnios sometimes means oesophageal atresia

### Elena Grechi, Maria Francesca Brambillasca, Alice Rocca, Stefania Vincenti, Angela Azzinari, Ilaria Frugnoli, Maria Siano, Cinzia Pittoni, Giovanni Traina

#### Division of Neonatology Department of Paediatrics, S.M delle Stelle Hospital, Melzo, Milan, 20060, Italy

##### **Correspondence:** Elena Grechi (grechi.elena@gmail.com)

**Background**

Oesophageal atresia (OA) encompasses a group of congenital anomalies comprising of an interruption of the continuity of the oesophagus with or without a persistent communication with the trachea. It is a relative common congenital malformation occurring in one in 2500 – 3000 live births. The majority of cases of OA are sporadic/non – syndromic.

**Case report**

B.V.J. born at 35+4 weeks of gestational by physiological birth. The birth weight was 2600 grams and polyhydramnios was noted during gestation. APGAR 10/10. During the first minutes of life, we note excessive salivation, so we pass a catheter through mouth into the oesophagus and it does not be able to descend more than 10 cm from mouth. In suspicious of OA, we perform an X – Ray that show the tip of the catheter arrested in the superior mediastinum (fig. 1).

The infant is placed in the incubator while monitoring the vital sign. We placed a suction catheter (10 French) in the upper oesophageal pouch to suction secretions and prevent aspiration. We provide a vascular access and start intravenous fluid administration. The acid/base equilibrium is adequate (pH 7.26, pCO2 51, lact 4.2, BEB -5.0) and the blood sample shows negative infection index. The baby has a good breath, he has some brief apnoea that solve autonomously. We alert the emergency transport and the newborn is transported to UTIN and receives a surgical correction in 2^nd^ day of life.

**Conclusion**

The OE is a relative common malformation and OA with distal tracheooesophageal fistula is the most common variety (86%). The diagnosis of OA may be suspected prenatally by the finding of a small or absent foetal stomach bubble on ultrasound scan performed after the 18th week of gestation. Polyhydramnios alone is a poor indication of oesophageal atresia (1%) but the newborn infant of a mother with polyhydramnios should always have a nasogastric tube passed soon after delivery to exclude OA. Infants with OA are unable to swallow saliva and are noted to have excessive salivation requiring repeated suctioning, but also respiratory distress, feeding difficulties, choking, and frothing in the first few hours of life. The etiology of OA is likely to be multifactorial and remains unknown. Over 50% of infants with OA have one or more additional anomalies. OA requiring surgical repair. Although mortality rates associated with this procedure are low, children may go on to have gastrointestinal and respiratory complications throughout childhood.

Informed consent to publish has been obtained from the parents.

**Fig. 1 (abstract A8).**
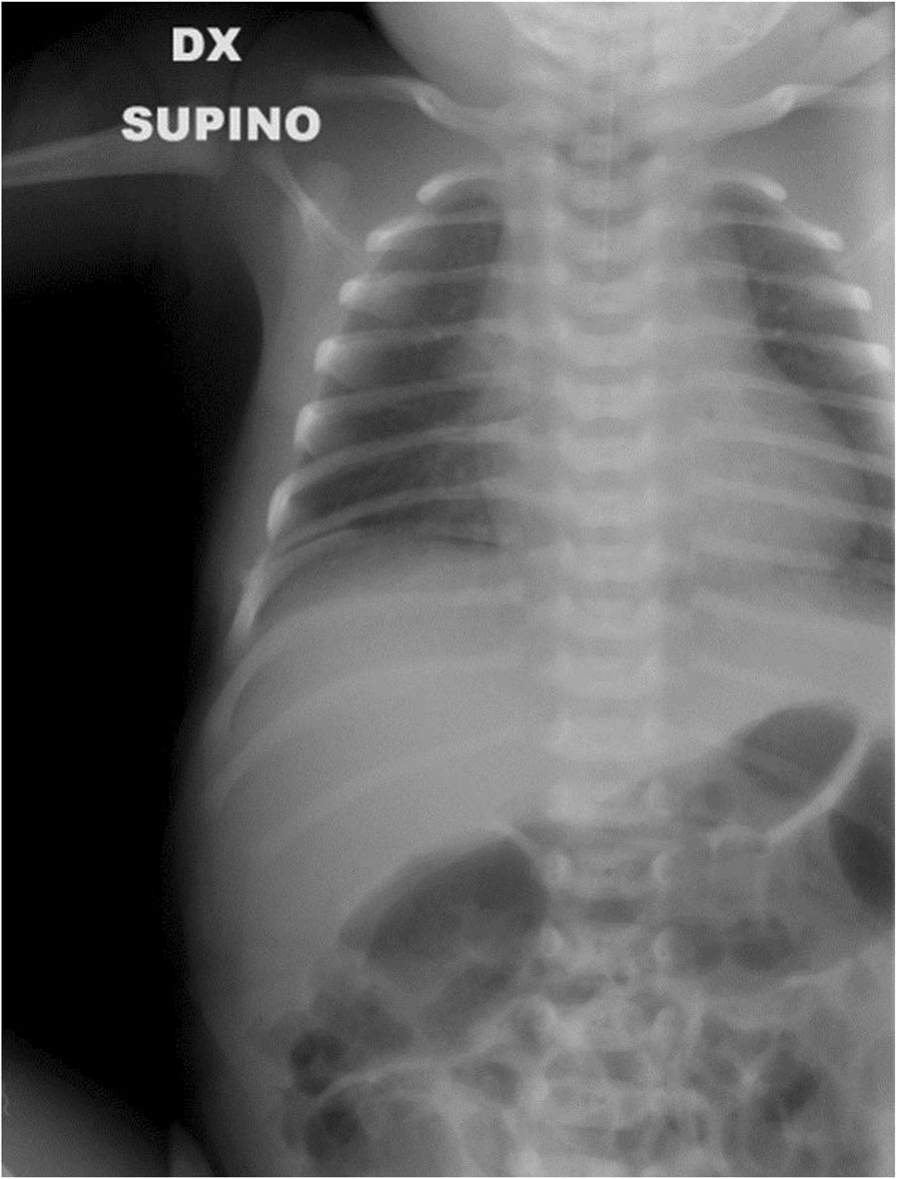
The tip of the catheter arrested in the superior mediastinum

## A9 Religion: Breaking reason or opportunity to improve?

### Giovanna Leone, Carmela Serlenga, Roberta Maffioli, Cristina Bellan

#### ^**1**^Neonatal Intensive Care Unit, TIN ASST-Bergamo EST, Ospedale Bolognini Seriate, Italy

##### **Correspondence:** Giovanna Leone (giannaleone@yahoo.it)

**Background**

Anaemia of Prematurity (AOP) is a pathological condition unlike physiologic anaemia in newborns The pronounced decline in the haemoglobin (Hb) concentration that occurs in ELBW infants is usually associated with abnormal clinical signs and requires allogeneic RBC transfusions AOP is characterized by reduced endogenous erythropoietin (EPO), reduced RBC lifespan and hypo-regenerative bone marrow Non-physiologic factors related to prematurity, such as phlebotomy losses for laboratory evaluations and infections resulting in oxidative haemolysis, also contribute to high transfusion preterm infants. [1-4]

**Case report**

M.J.R. was born at 24 weeks of G.E. and a birthweight of 630 g from Jehovah's Witnesses parents. Histologic examination of the placenta showed severe chorionamnionitis and positive placental swabs for serratia marcescens. The infant had hyaline membrane disease that required prolonged ventilatory assistance, symptomatic patent ductus arteriosus, IVH I grade, severe anemia. The parents did not consent to the administration of blood products. The ordinary judge was informed of their decision. Blood sampling was minimized and non-invasive blood-gas monitoring was used consistently. It was agreed with the parents to start treatment with recombinant erythropoietin as soon as the trophism allowed. The critical conditions and severe anemia required the administration of two hemotransfusions in the first two weeks of life. At the 30th weeks it was possible to start therapy with erythropoietin, together with martial integration, prevented further transfusions. Taking care of JR began with that of their parents. At their request they were accompanied to neonatal intensive care unit by their spiritual guide. The problems related to such premature birth were illustrated to them. They explained the reasons for their refusal, were informed that, while understanding and respecting their point of view if the clinical conditions required it, we could not be exempt from administering blood products to their baby; were reassured that all measures to minimize blood loss had been implemented pending the start of erythropoietin therapy.

**Conclusions**

The religious beliefs of the parents with could become grounds for comparing proved to be a source of enrichment and improvement .

Informed consent to publish has been obtained from the parents.

**References**

1. Serdar Alana Saadet Arsanb. Prevention of the anaemia. International Journal of Pediatrics and Adolescent Medicine Volume 2, Issues 3–4, September–December 2015, Pages 99-106.

2. S. Antoncecchi, A.M. Casadei, A. Del Vecchio, G. Girelli, P. Isernia, M. Motta, D. Regoli, C. Romagnoli, G. Tripodi, C. Velati. Raccomandazioni per la terapia trasfusionale in neonatologia.

3. Vamvakas EC, Strauss RG. Meta-analysis of controlled clinical trials studying the efficacy of rHuEPO in reducing blood transfusions in the anemia of prematurity. Transfusion 2001 Mar; 41(3):406-15.

4. Aher SM, Ohlsson A. Late erythropoiesis-stimulating agents to prevent red blood cell transfusion in preterm or low birth weight infants. Cochrane Database Syst Rev. 2019 Feb 15; 2:CD004868. Epub 2019 Feb 15.

## A10 Double Aortic Arch in a newborn: a case report

### Gianluca Lista^1^, Savina Mannarino^2^, Silvia Bianchi^1^, Luisa Federica Nespoli^2^, Giuseppina Mancini^1^

#### ^1^Department of NICU, Ospedale V. Buzzi, Milano, Italy; ^2^Department of Pediatric Cardiology, Ospedale V. Buzzi, Milano, Italy

##### **Correspondence:** Gianluca Lista (gianluca.lista@asst-fbf-sacco.it)

**Background**

The double aortic arch is a rare anomaly of the aortic arch. The mature aortic arch is formed through a process of appearing and involution of six paired primitive aortic arches in a cranial to caudal order. In this model some of the primitive arches regress, whereas others persist and develop, resulting in the normal left aortic arch and left descending aorta. Anomalies in this process of involution may result in various anomalies of the aortic arch. Congenital variants and anomalies of the aortic arch are important to recognize because they may be associated with vascular rings or other complex congenital heart diseases. A vascular ring is formed when trachea and esophagus are completely surrounded by large vessels or atretic segments, with possible airway and/or esophageal compression and appearance of breathing difficulties, stridor and/or dysphagia. A double aortic arch is the most common cause of a symptomatic vascular ring.

**Case Report**

We describe the case of a newborn (G.N) born at 40 weeks by vaginal delivery, birth weight 3765g. Regular course of pregnancy; Apgar score 1 ’= 9, 5’ = 9. During the first day of life, G.N. developed respiratory distress and stridor requiring High Flow Nasal Cannula for six days. The chest X-Ray and otorhinolaryngologist evaluation, performed because of the persistence of the symptomatology, were normal. The electrocardiogram revealed isolated extrasystolia. The echocardiography raised the suspicion of an aortic arch anomaly, with just two epiaortic vessels visualized. Further investigations with CT-angiography showed a double aortic arch and a complete vascular ring, with left carotid and subclavian artery originating from left aortic arch and right carotid and subclavian artery originating from right aortic arch. The vascular ring determined a pronounced compression of trachea and esophagus. During the hospitalization, his general conditions gradually improved, with regular growth, good feeding tolerance and slight inspiratory stridor. He underwent surgical correction at two months of life. There was a noncomplicated postsurgical course.

**Conclusions**

Double aortic arch is the most common cause of a symptomatic vascular ring. Signs and symptoms like respiratory distress without any other cause, inspiratory stridor and/or dysphagia may be the first clinical presentation of this rare pathology. Echocardiography raises the suspicion of the diagnosis, which is then confirmed by CT or MR angiography. Surgical repair is needed if there is compression of vital structures and the postsurgical course is general uncomplicated.

Informed consent to publish has been obtained from the parents.

## A11 Neonatal alloimune neutropenia: an unexpected finding in healthy term infants

### Chiara Giovanettoni, Valeria Manfredini, Anna Pirelli, Salvatore Barberi

#### Neonatal intensive Care Unit, Azienda Ospedaliera “ASST Rhodense”, P.O. Rho, Italy.

##### **Correspondence:** Chiara Giovanettoni (giovanettoni.chiara@gmail.com)

**Background**

Neutropenia is a frequent condition in preterm and critically-ill neonate. We describe two cases of neonatal alloimmune neutropenia (NAIN) in well appearing term infants.

**Case report**

O. A. was born from a pregnancy complicated by gestational diabetes. The family history was negative. Vaginal swab was positive for a β-haemolytic streptococcus B and complete antibiotic prophylaxis was provided during delivery. Because low for gestational age (2900 gr, 3°-10° ple), blood tests were performed showing low white blood cell count (5180 cells/mm3) and severe neutropenia (ANC-minimum value 40 cells/mm3), asymptomatic. Antibiotic therapy was started, with no ANC increase. Microbiological tests showed negative and treatment was suspended after repeated CRP proved negative. Finally, auto-antibodies against neutrophils were screened, resulting positive. Molecular typing for the antigenic HNA system showed a mismatch between the baby and his mother with reference to the HNA-1b antigen (inherited from the father), concluding for a NAIN caused by anti HNA-1b maternal antibodies.

T.A. was born from precipitous labour in uncomplicated pregnancy. The amniotic fluid at birth was meconium-stained, malodorous. At birth Apgar score was 9/10 and baby’s weight adequate for gestational age. Blood tests were performed because of the amniotic fluid, showing increased CRP (2.53 mg/dL) and WBC count (8930/mm3) with reduced absolute neutrophil count (ANC 1%, 140 cells/mm3). The baby received antibiotic treatment with ampicillin plus netilmicin for 7 days, with no increase in the ANC. Auto-antibodies against neutrophils were screened, showing positive. Molecular typing for the antigenic HNA system showed a mismatch between the baby and his mother with reference to the HNA-1a antigen (inherited from the father), concluding for a NAIN caused by anti HNA-1a maternal antibodies.

In both cases, treatment with subcutaneous granulocyte-colony stimulating factor (Filgrastim 10 mc/kg/day) twice a day was started, allowing the rapid increase of the ANC after the 2nd dose. During the next follow-up, the value of the ANC remained in the normal range.

**Conclusion**

Neutropenia affects up to 8% newborns in the intensive care setting. The clinical presentation of NAIN is variable. Sepsis, omphalitis and temperature instability has been described, however infection is not observed in as many as 40% infants with NAIN. [1, 2] Our reports supports previous data showing NAIN is often incidentally observed on blood tests rather than in the presence of overt infection. We may then suppose it is underestimated in well appearing children. Thus, in case of persistent neutropenia, an immune-mediate etiology involving anti-neutrophil antibodies should be screened.

Informed consent to publish has been obtained from the parents.

**References**

1. Maheshwari A. Neutropenia in the newborn. Curr Opin Hematol. 2014; 21:43-49.

2. Del vecchio A. Christensen A.D. Neonatal neutropenia: what diagnostic evaluation is needed and when is treatment recommended? Early Hum Dev. 2012. 88S2: S19-S24.

## A12 Lethal hypertrophic cardiomyopathy in a neonate with Noonan syndrome

### Alessandra Mayer^1,2^, Marco Colombo^2^, Gaia Francescato^1^, Federico Schena^1^, Benedetta Beltrami^3^, Maria F Bedeschi^3^, Lucia Mauri^4^, Anna M Colli^4^, Marco Papa^4^, Beatrice L Crippa^1,2^, Ilaria Amodeo^1,2^, Fabio Mosca^1,2^

#### ^1^ Foundation IRCCS Ca’ Granda Ospedale Maggiore Policlinico, NICU, Milan, Italy; ^2^ University of Milan, Department of Clinical Sciences and Community Health, Milan, Italy; ^3^ Foundation IRCCS Ca’ Granda Ospedale Maggiore Policlinico, Medical Genetic Unit, Milan, Italy; ^4^ Foundation IRCCS Ca’ Granda Ospedale Maggiore Policlinico, Cardiology Unit, Milan, Italy

##### **Correspondence:** Alessandra Mayer (alessandra.mayer@unimi.it)

**Background**

Noonan syndrome (NS) is an autosomal dominant disorder with a prevalence of 1 in 1000–2500. It is included in the so-called RASopathies, a group of genetic syndromes caused by a mutation in the Ras/MAPK pathway. A mutation in PTPN11 gene on chromosome 12 can be identified in approximately 50% of cases, but other genes are involved in NS (e.g. KRAS, SOS1, NRAS and RAF1). Congenital heart diseases (CDHs) are often associated, in particular pulmonary stenosis (PS) and hypertrophic cardiomyopathy (HCM). The clinical presentation and evolution of the latter is highly related to the underlying genetic mutation.

**Case report**

An infant was born by caesarean section at term after a pregnancy complicated at 35 weeks by the detection of polyhydramnios and both abdominal and pleural effusion, treated *in utero* by placing a thoracoamniotic shunt. At birth the infant presented severe respiratory distress, requiring tracheal intubation and chest tube insertion. Echocardiography showed a biventricular HCM (Figure 1) with multivalvular dysplasia (in particular PS), while electrocardiography revealed hyper-right axis deviation, which were consistent with Noonan syndrome. Whole exome sequencing identified heterozygous pathogenic variant p.Asn308Thr in PTPN11 gene and confirmed diagnosis. During the first weeks of life the infant presented frequent premature ventricular beats and several episodes of sustained ventricular tachycardia, which were controlled with high doses of beta blocker therapy. Nevertheless, clinical picture worsened over time with progressive biventricular outflow tract obstruction and severe diastolic dysfunction. The patient died from congestive heart failure at 3 months of age.

**Conclusion**

Up to 90% of patients diagnosed with NS have cardiovascular defects. The most common is PS, followed by HCM and atrial septal defects. HCM is the most common cardiac defect in patients with mutations in the RAF1 gene, being less common in PTPN11-mutated patients. HCM in NS has a worse prognosis compared to idiopathic or familial HCM and it is associated with a higher incidence of other concomitant CHDs. Our case confirms the poor prognosis association of PS and severe obstructing HCM secondary to pathogenic variant p.Asn308Thr of PTPN11 gene: conversely to isolated PS, in fact, in this case no effective surgical or percutaneous therapeutic option can be considered.

Informed consent to publish has been obtained from the parents.

## A13 A blueberry muffin baby: don’t forget the bone marrow

### Grazia Morandi, Paola Sindico, Elisa Agazzani, Simona Boccacci, Gabriella Calzetti, Silvia Orlandini, Valeria Angela Fasolato

#### Neonatal Intensive Care Unit, “C. Poma” Hospital, ASST of Mantova, Mantova, Italy.

##### **Correspondence:** Grazia Morandi (grazia.morandi@asst-mantova.it)

**Background**

“Blueberry muffin baby” was initially coined to describe cutaneous manifestations of rubella infection observed in neonates during the American epidemic of the 1960s. Nevertheless, the typical skin lesions may also be present in rare neonatal malignancy and hematologic.

**Case report**

This is the case of a full-term boy presented soon at birth with purple, erythematous, oval or circular macules and papules, widespread in particular on the trunk, head, and neck; some of those developed in the following days a petechiae-like appearance and others a central ulceration and a crust (Fig. 1 and Fig. 2). The baby was apparently well, with normal respiratory and heart parameters, he had fever in the second day of life (T 38°C), resolved with paracetamol. We performed blood samples, finding an increased C-Reactive Protein (CRP) (115.3 mg/L), a white blood cells (WBC) count of 6670/mm3 (Neutrophils 5400/mm3, Lymphocytes 1270/mm3), and normal liver and kidney functions. TORCH infections were excluded both in the mum and in the neonate. After blood, liquor and urine cultures, he was started with a double antibiotic therapy. After 48 hours, CRP was decreasing (66.8 mg/L), but WBC count revealed an important immunodeficiency, with a very low number of lympocytes (WBC 3610/mm3, N 2480/mm3, L 550/mm3). Because of the “blueberry muffin rash” and the marked lymphopenia we hypothesized a bone marrow disorder, in particular the Langerhans cell histiocytosis. Our suggestion was confirmed in the Paediatric Hematology and Oncology department of the hospital where we transferred the baby. There a peripheral blood smear, a bone marrow aspirate, a skin biopsy and then a total body CT-scan revealed a multisystem neonatal onset of this rare condition.

**Conclusion**

Historically, neonatal blueberry muffin lesions were associated to congenital infections, such as the TORCH complex (toxoplasmosis, other, rubella, cytomegalovirus, herpes) but they denote the expression of an extramedullary hematopoiesis, present also in rare hematologic dyscrasias, which must be taken into account in the differential diagnosis. [1-5]

Informed consent to publish has been obtained from the parents.

**References**

1. Mehta V, Balachandran C & Lonikar V. Blueberry muffin baby: A pictoral differential diagnosis. Dermatol Online J. 2008 Feb 28;14(2):8.

2. Allen CE, Ladisch S, McClain KL. How I treat Langerhans cell histiocytosis. Blood 2015; 126 (1): 26–35.

3. Walkovich K, Connelly JA. Primary immunodeficiency in the neonate: Early diagnosis and management. Semin Fetal Neonatal Med. 2016;21(1):35–43.

4. Pettinger KJ, Solman L, Mathew B, et al Cutaneous Langerhans cell histiocytosis presenting in a neonate Archives of Disease in Childhood 2018;103:993

5. Lasek-Duriez A, Charkaluk ML, Gosset P, Modiano P. Histiocytose langerhansienne congénitale et « Blueberry Muffin Baby » [Blueberry Muffin Baby and Langerhans' congenital cell histiocytosis]. Ann Dermatol Venereol. 2014;141(2):130–133.

**Fig. 1 (abstract A13).**
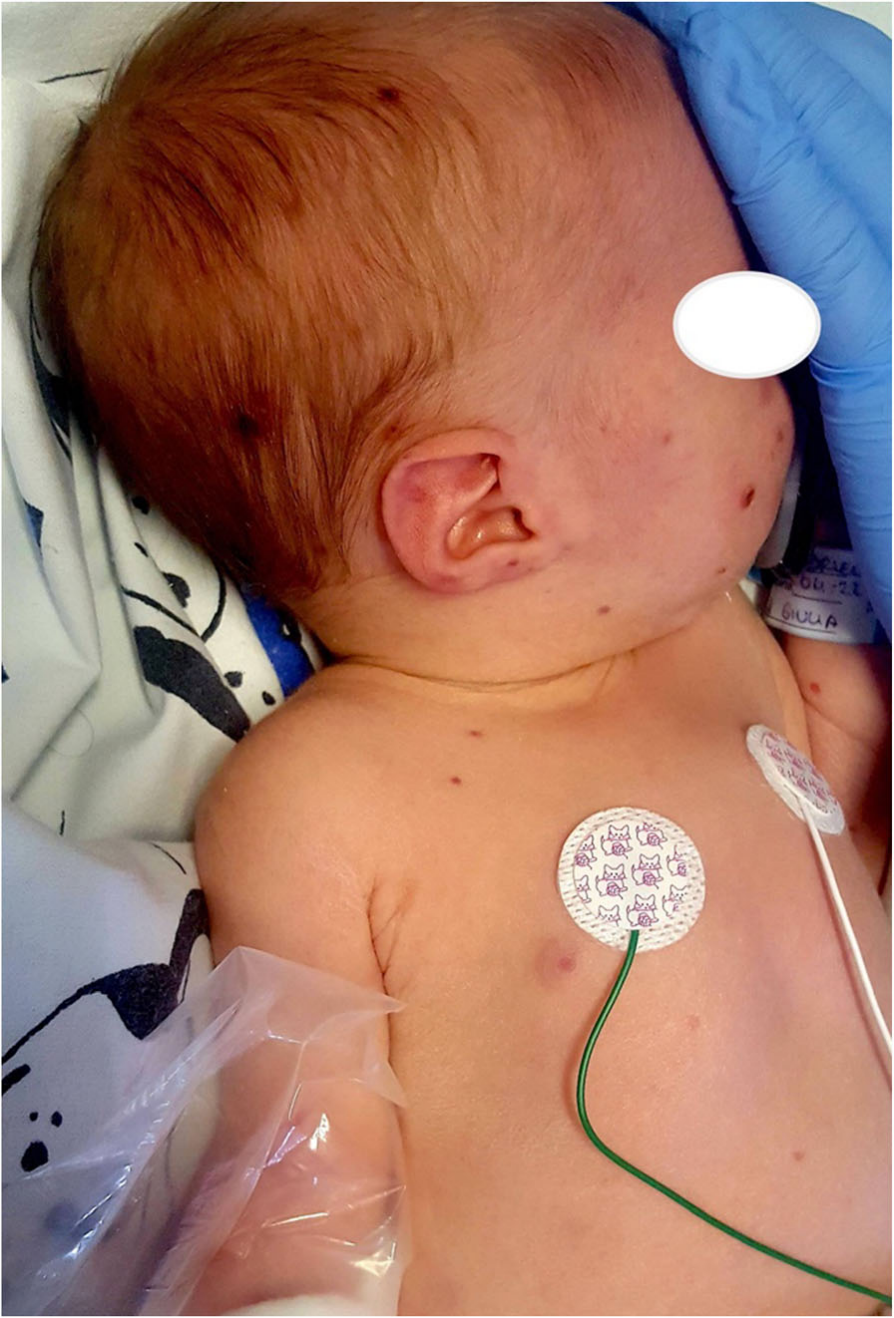
Purple, erythematous, oval or circular macules and papules at birth

**Fig. 2 (abstract A13).**
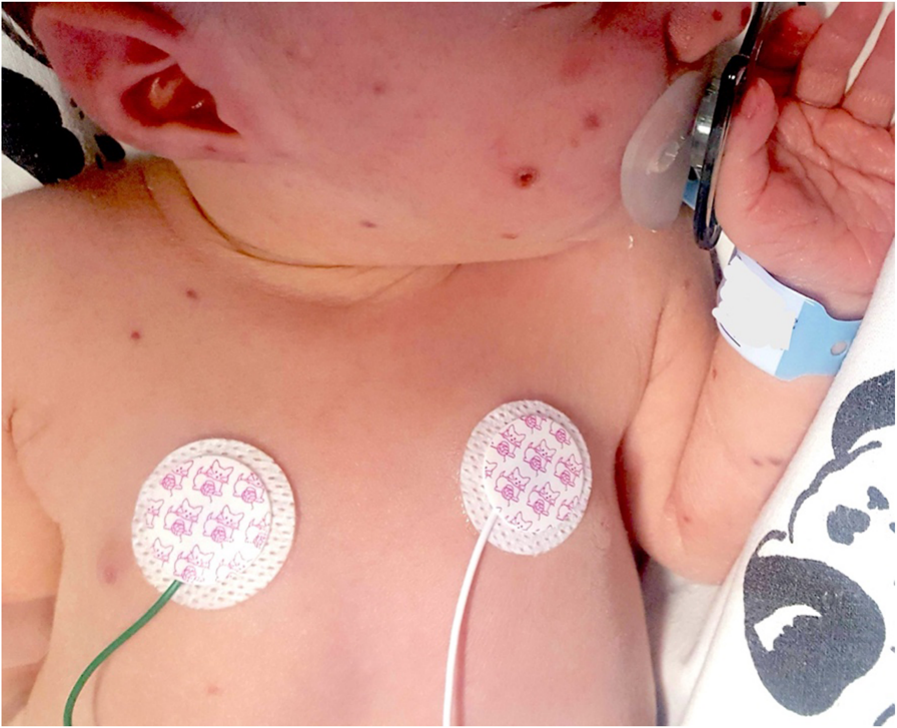
Development of the rash with some lesions with a central ulceration and a crust and others with a petechiae-like appearance

## A14 Aplasia cutis congenita or congenital Volkmann syndrome: the clue is in the bone

### Grazia Morandi, Paola Sindico, Elisa Agazzani, Simona Boccacci, Silvia Orlandini, Francesca Paola Fusco, Ilaria Lombardo, Giulia Vellani, Valeria Angela Fasolato

#### Neonatal Intensive Care Unit, “C. Poma” Hospital, ASST of Mantova, Mantova, Italy.

##### **Correspondence:** Grazia Morandi (grazia.morandi@asst-mantova.it)

**Background**

Aplasia cutis congenita (ACC) is a heterogeneous group of conditions characterized by the congenital absence of epidermis, dermis, and in some cases, subcutaneous tissues. It is a rare disease, with an estimated incidence of 3 in 10,000 births; it involves more frequently the scalp, but almost everybody surface may be interested; ACC can occur as an isolated defect or associated with other congenital anomalies such as limb anomalies or embryologic malformations: bone defects occurs in approximately 20% of cases. The classification, proposed by Frieden in 1986, is still accepted today, providing 9 groups of ACC. [1]

**Case report**

This is the case of a full-term girl presented with a 3-cm x 4-cm yellow ulcerated skin area on the right forearm, associated to hypotrohy and hyporeflexia of the same forearm and hand (Fig. 1 and Fig 2). She was born from an uncomplicated pregnancy, with no history of maternal trauma or infection, no drug use, or chimica exposure. Prenatal ultrasounds were normal. Family history was also negative for blistering disorders. The arm X-ray showed at the same level of the skin lesion, a clear line of increased bone density both on radius and ulna, revealing a site of a possible insult, occurred during pregnancy and characterized by an increased localized pressure, causing muscular ischemia, nerve damage, tissue necrosis, and fibrosis (Fig. 3). A multidisciplinary treatment was chosen: daily guaze dressings with fucidin ointment and petroleum jelly were associated to physiotherapy to improve the use of the arm and of the hand. According to Frieden’s classification we may define this case as an aplasia cutis congenita type VII or taking into account the muscular and neurological impairment as well as the radiological finding as a case of congenital Volkmann syndrome, caused by an amniotic band. [2-4]

**Conclusion**

ACC is a rare congenital condition, which may present as solitary or multiple lesions and can appear on any part of the body. The diagnosis is typically made from clinical examination, withholding the lesional biopsy, given the patient's age. Besides radiological findings must be considered of pivotal importance to make the right diagnosis, to check the involvement of deep tissues and organs, as we saw in our case.

Informed consent to publish has been obtained from the parents.

**References**

1. Brackenrich J, Brown A. Aplasia Cutis Congenita. [Updated 2019 Nov 8]. In: StatPearls [Internet]. 2) Droubi D, Rothman IL. Aplasia cutis congenita of the arm with associated radial dysplasia: case report, review of the literature, and proposed classification. Pediatr Dermatol. 2014 May-Jun;31(3):356-9.

2. Nagore E, Sanchez-Motilla JM, Febrer MI et al. Radius hypoplasia, radial palsy, and aplasia cutis due to amniotic band syndrome. Pediatr Dermatol 1999;16:217–219.

3. Neri I, Magnano M, Pini A, Ricci L, Patrizi A, Balestri R. Congenital Volkmann Syndrome and Aplasia Cutis of the Forearm: A Challenging Differential Diagnosis. JAMA Dermatol. 2014;150(9):978–980.

4. Agrawal H, Dokania G, Wu SY. Neonatal volkmann ischemic contracture: case report and review of literature. AJP Rep. 2014;4(2):e77-80.

**Fig. 1 (abstract A14).**
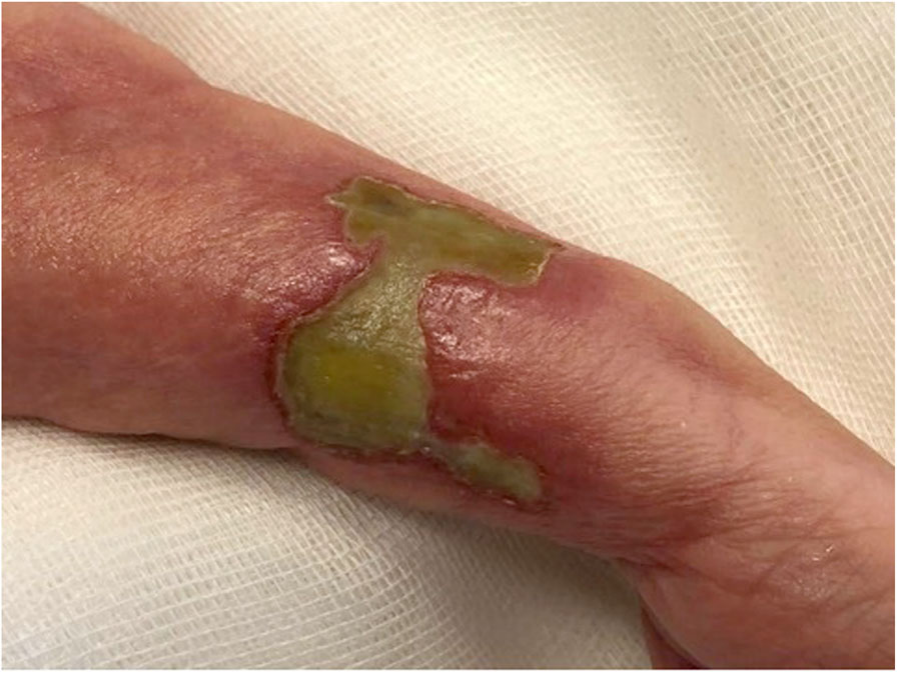
Well-defined margins, aplasia cutis of the right arms

**Fig. 2 (abstract A14).**
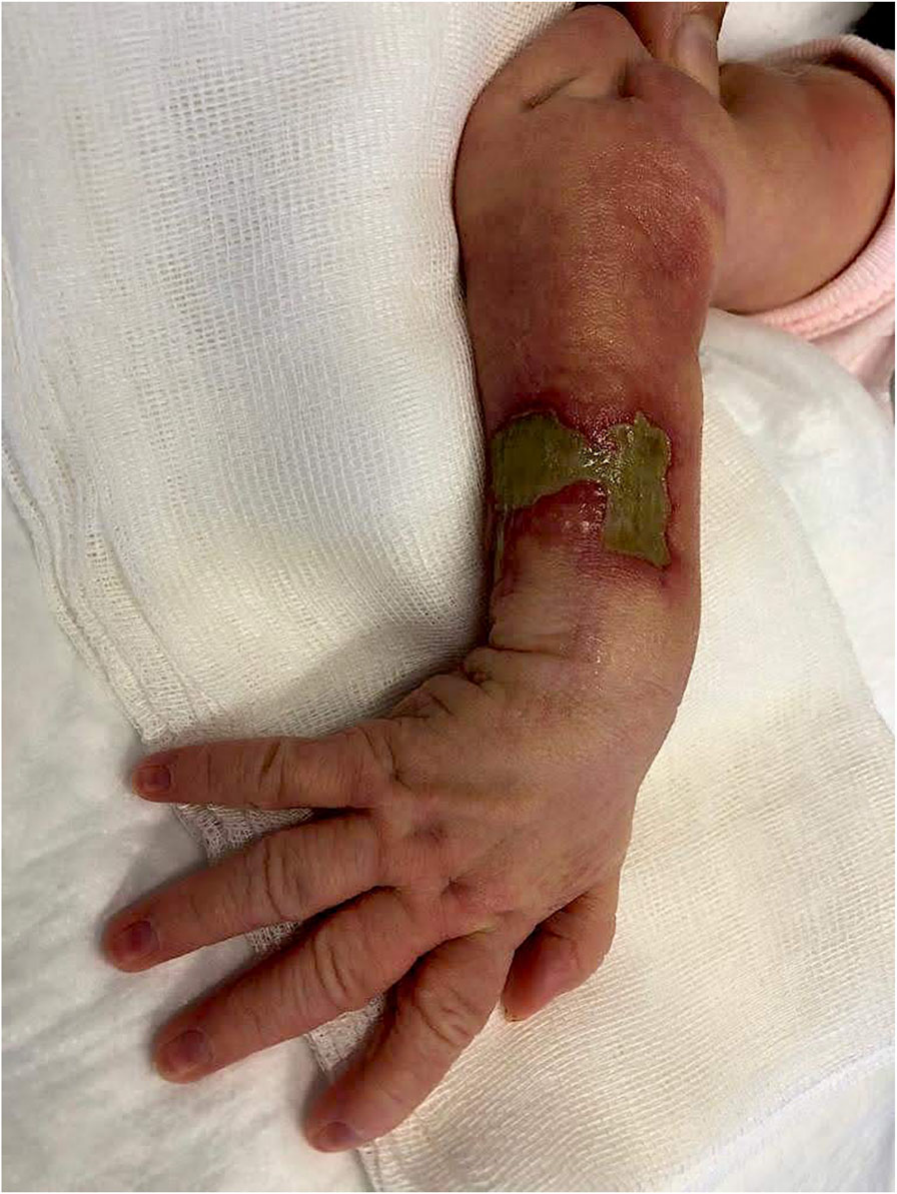
Erythematous aplasia cutis and hypotrohy of the right arms

**Fig. 3 (abstract A14).**
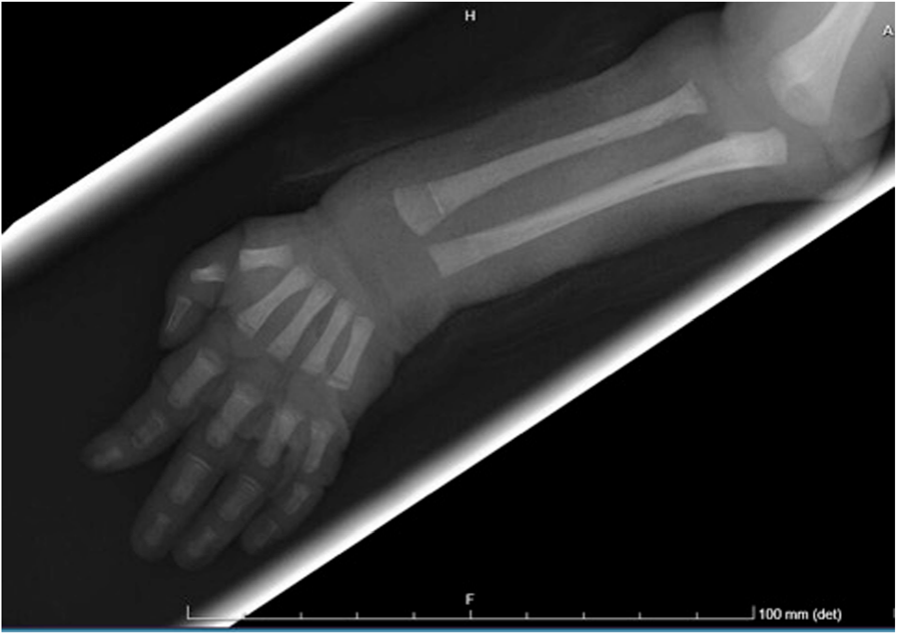
X ray of the right forearm, showing the white lines on both radius and ulna

## O1 Nurse care for the neonatal thermoregulation in NICU

### Raffaella Lucchini, Marilena Ferraresi, Azzurra Saggiorato, Grazia Morandi, Paola Sindico, Simona Boccacci, Silvia Orlandini, Valeria Angela Fasolato

#### Neonatal Intensive Care Unit, “C. Poma” Hospital, ASST of Mantova, Mantova, Italy.

##### **Correspondence:** Raffaella Lucchini (raffaella.lucchini@asst-mantova.it)

**Background**

As The World Health Organization reminds “adequate environmental warmth is essential in the care of small infants because they could not maintain their own body heat”. The normal temperature of a neonate ranges from 36.5 °C - 37.5 °C. Hypothermia has been recognized as a significant contributor to neonatal morbidity and mortality for all newborn infants, and has been described on every continent and even in many countries that are considered warm, as Italy.

**Materials and Methods**

We analyzed the temperature of all the neonates admitted to the Neonatal Intensive Care Unit in 2019, till November. Exclusion criteria was therapeutic hypothermia. To study the best approaches to maintain the right neonatal temperature we organized meetings, reviewed the literature and finally we drew up the following department rules: - the nurse prepares radiant warmers in advance in the delivery room - plastic wrap and hat are always used under the 32 gestational weeks (GW) - the nurse check the room temperature in NICU, which must be draught-free and around 25°C - only warmed humidified gases were employed both during the resuscitation and in NICU - in every incubator or neonatal bed a warm nest around the baby was shaped with plastic single use clothes to reduce the temperature losses - humidity and temperature of the incubator following the tables for the different GWs have been inserted in the nurse folder.

**Results**

We collected 294 admission temperatures: the mean body temperature was 36.7°C; 66.1% (198 out of 294) had a T°C between 36.5-37.5°C; 25.9% (74 out of 294) between 36-36.5, 5.0% (5 out of 294) under 36 and 3.0% (9 out of 294) above 37.5°C. Missing data were around 5%. February, August and November were the months where temperatures under 36°C were higher (13.3, 11.1, 10.3% respectively), probably because of the outside whether, colder during winter and because of the air conditioning during summer time. (fig.1)

**Conclusions**

From Budin’s 1907 publication of The Nursling till the last 2015 Neonatal resuscitation guidelines it has long been recognized that the admission temperature of newly born non-asphyxiated infants is a strong predictor of mortality at all gestational ages. Besides, it is mandatory to develop nurse strategies to keep warm babies in NICU and it is important to collect department data to improve nurse neonatal care.

**Fig. 1 (abstract O1).**
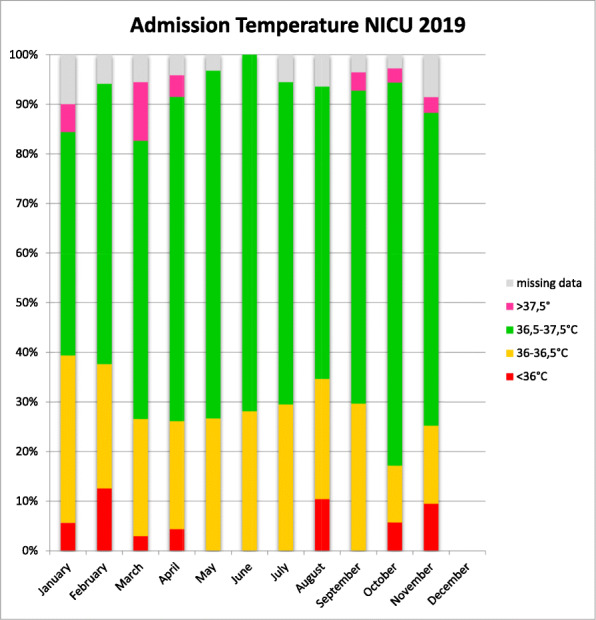
Admission temperatures in NICU in 2019

## A15 Skin-to-skin: tools to make it feasible and safe

### Raffaella Lucchini, Martina Perdomini, Marilena Ferraresi, Azzurra Saggiorato, Grazia Morandi, Paola Sindico, Elisa Agazzani, Valeria Angela Fasolato

#### Neonatal Intensive Care Unit, “C. Poma” Hospital, ASST of Mantova, Mantova, Italy

##### **Correspondence:** Raffaella Lucchini (raffaella.lucchini@asst-mantova.it)

**Background**

Skin-to-skin contact (SSC) between a newborn infant and its mother has well-documented benefits and it is recommended by all major organizations responsible for the well-being of newly born infants, including The World Health Organization (WHO), the American Academy of Pediatrics (AAP), the Academy of Breastfeeding Medicine (ABM), and the Neonatal Resuscitation Program (NRP); it improves physiologic stability for both mother and baby, increases maternal attachment behaviours, protects against the negative effects of maternal–infant separation, supports optimal infant brain development and promotes initiation breastfeeding. However, it could be dangerous if midwives and nurses do not cooperate to check mother and child vigilance.

**Material and methods**

To promote skin-to-skin soon after birth we shared the supporting literature and discussed benefits; meetings and lessons for nurses, midwives, neonatologists and gynaecologists were organized, a inter-department monitoring form was prepared to check the child during skin-to-skin and parents’ information leaflets were given. (Fig.1)

**Results**

Since July, 372 children born from vaginal delivery (VD) and 181 born from caesarean section (CS) were examined: respectively the 90% (329 out of 363) and the 89% (140 out of 158) underwent the skin-to-skin contact with the mother for 2 hours soon after birth. In the VD group 23% (8 out of 35) of the children didn’t do SSC because of neonatal problems, 40% (14 out of 35) because of mother problems and 37% (13 out of 35) because of department problems; in the CS group 38.5% (7 out of 18) didn’t do SSC because of neonatal problems, 17% (3 out of 18) because of mother problems and 44.5% (8 out of 18) because of department problems. Moreover 60 children in the first group and 32 in the second one interrupted SSC during the 2 hours: child problems were present in the 18.5% (11 out of 60) and in the 19% respectively (6 out of 32%); mum problems in the 38.5% (23 out of 60) and 37.5% (12 out of 32%) of the children; department and organization problems occurred in the 43.5% (14 out of 32) and in the 43.5% of the two groups.

**Conclusion**

Immediate skin to skin contact between mother and newborn is supported in the literature improving neonatal thermoregulation, breastfeeding and bonding. The policy of a baby-friendly institution, according to UNICEF guidelines, must promote SSC and record data about it to overcome any obstacle, which prevent the success of this natural practice.

**Fig. 1 (abstract A15).**
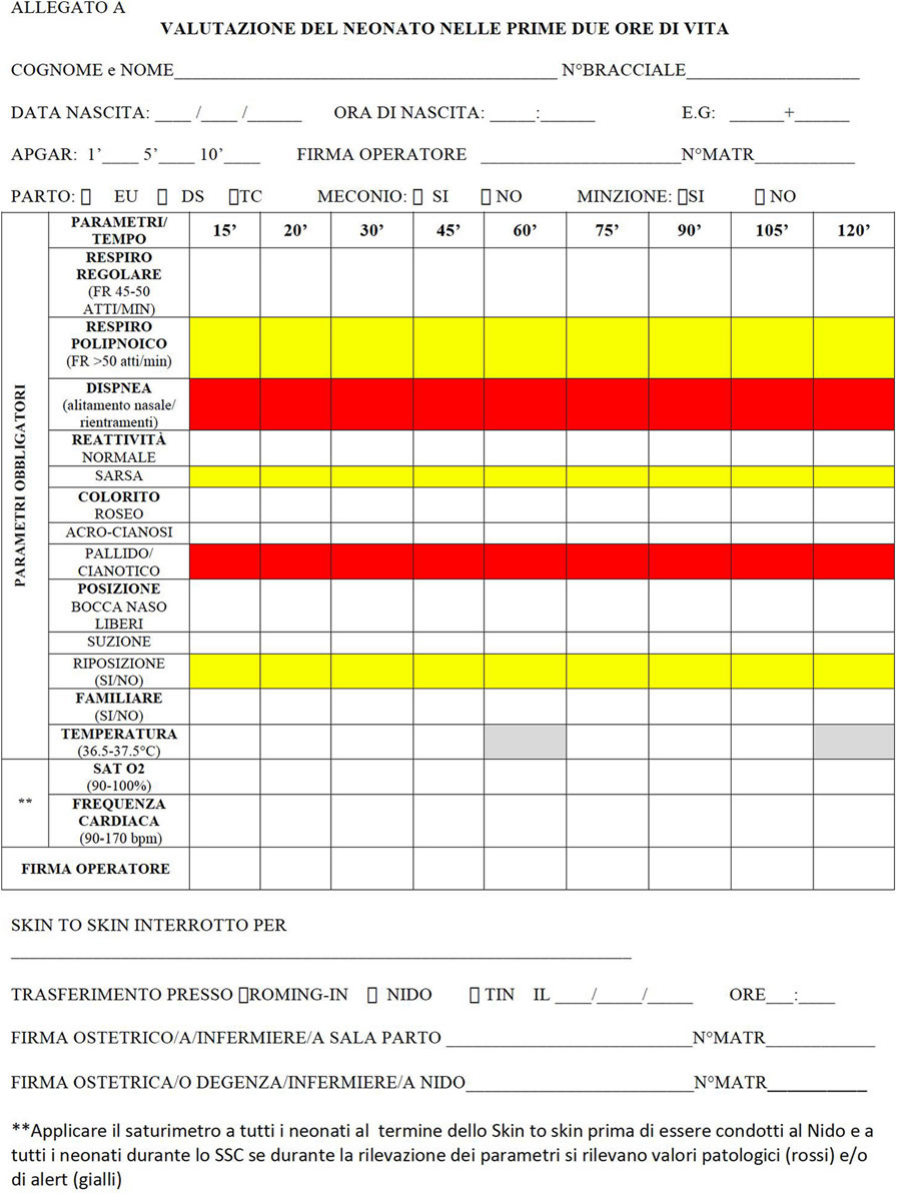
Skin-to-skin format in our department

## O2 Case report: : newborn with congenital melanocytic nevi

### Federica Nociforo, Gianna Leone, Monica Airoldi, Cristina Bellan

#### NICU and Neonatology, ASST-Bergamo Est, Seriate (BG), Italy

##### **Correspondence:** Federica NociforoFederica Nociforo (federica.nociforo@asst-bergamoest.it)

**Background**

Congenital Melanocytic Nevus (GCMN) is a pigmented skin lesion with a slight predominance in female. Its incidence is estimated in <1:20,000 newborns[1]. Usually confined to the skin, but rarely invade the underlying fascia, muscle, or further deep structures. The estimated life-time risk for malignancy (cutaneous and noncutaneous), is 4.5-10%. GCMN have been described to be associated with spina bifida occulta, neurofibromatosis, lipomatosis, and various other disorders. GCMN are caused by malformations of the neuroectoderm and occasionally neural elements, following de-regulated growth and arrest of melanocytes during migration from the neural crest to the skin [2]. The diagnosis is clinical. MRI and neurological evaluation are useful for identifying complications. Differential diagnoses include small cell melanoma, rhabdoid melanoma, and melanoma with divergent RMS differentiation. A paradominant mode of transmission is hypothesized (a postzygotic mutation that occurs at an early stage of development and causes a somatic loss of heterozygosity).

**Case report**

B.E. inborn, 37w+5gg, eutocic birth, firstborn of unrelated parents. APGAR: 9/10. LGA. Obstetric history: gestational diabetes controlled by diet. Negative family history. At birth diagnosis of Giant (GCMN) extending from the thorax to the thighs with satellite lesions to the limbs (figures 1 and 2). She is admit to TIN for the necessary checks. Cerebral ultrasound, EEG, FO, echocardiography, abdominal ultrasound, AABR are normal. Brain and spine NMR with and without contrast medium show a small area of hyperintensity in T1 and in T2 in the left amygdaloid region and analogous area with a minimally expansive effect in the anterior pontine right, as well as likely parenchymal melanosis; some small spots in the bilateral cerebellar superficial cortical area. Brain NMR, after 6 months, shows posterior arachnoid cystic formation from D5 to D11 of about 66 mm in the endorachid area, which displaces the medullary cord a slightly anteriorly. Neurosurgical counseling ascribes this lesion as a cystic formation not associated with cutaneous lesions worthy of careful follow-up MRI for risk of spinal cord compression. Due to a possible malignant transformation, the dermatological follow-up of the nevus must be constant. GCMN requires a multidisciplinary approach and a scrupulous management for a careful follow up.

**Conclusion**

Our patient is in follow-up at our clinic in collaboration with the Pediatrician, the neurosurgery and dermatology clinic. Moreover, as already started during the hospitalization, a psychological support for the family has been started to better address this pathology.

Written informed consent for publication of their clinical details was obtained from parents of the patient.

**Fig. 1 (abstract O2).**
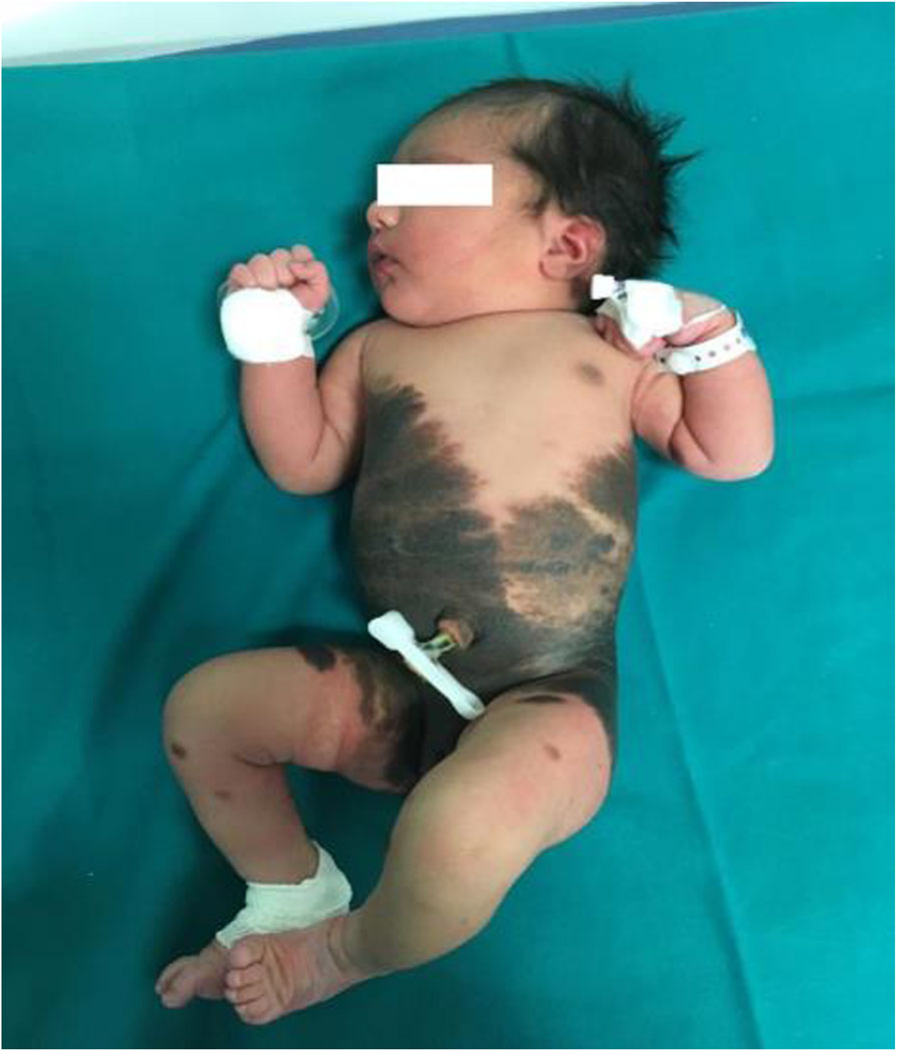
See text for description

**Fig. 2 (abstract O2).**
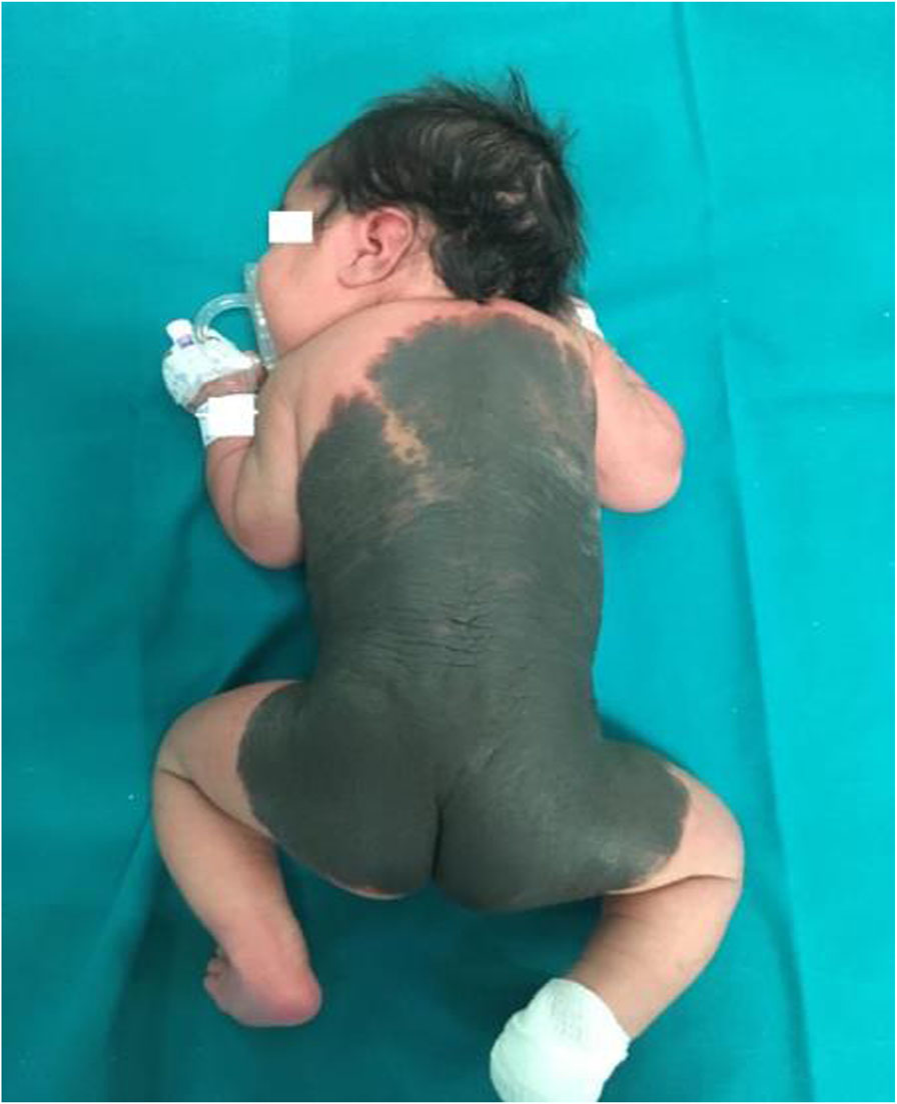
See text for description

**References:**

1. Viana AC, Gontijo B. Giant congenital melanocytic nevus. Bittncourt FV. An Bras Dermatol. 2013; Nov-Dec;88(6):863-78.

2. Escandòn-Pèrez S, Landeta-Sa AP. Giant congenital melanocytic nevi. 2019;76(6):251-258.

## O3 Spurious elevation of AST in a newborn due to a macroAST of maternal origin

### Laura Pogliani^1^, Erica Rampoldi^2^, Pierangelo Clerici^2,^ Benedetta Boldrighini^1^, Daniele Spiri^1^, Alberto Dolci^3^, Mauro Panteghini^3^

#### ^1^UO Pediatria, ASST Ovest Milanese, Legnano (MI) (Italy); ^2^UO Laboratorio Analisi, ASST Ovest Milanese, Legnano (MI) ) (Italy); ^3^Dipartimento di Scienze Biomediche e Cliniche “Luigi Sacco”, Università degli Studi di Milano ) (Italy)

##### **Correspondence:** Laura Pogliani (lauramaria.pogliani@asst-ovestmi.it)

**Background**

Macro-aspartate aminotransferase (macroAST) is a high molecular mass form of AST, formed by immunoglobulin binding to circulating enzyme, that reduces clearance and increases AST activity, leading to diagnostic confusion and unnecessary procedures. MacroAST should be considered a benign finding, widely described in adults and occasionally in children.

**Case Report**

A 3450 g Caucasian female neonate (M) was born at 40+5 weeks of gestation by vaginal delivery. Apgar scores at 1 and 5 minutes after birth were 9 and 10. Since the last delivery, 4 years before, the mother had isolated AST elevation, without elevation of ALT. She was investigated by liver and heart ultrasound and blood testing for viral, metabolic and autoimmune hepatic diseases without finding any abnormality. Hemolytic, muscular and myocardial causes of elevated AST activity were excluded. Polyethylene glycol (PEG) precipitation test and AST isoenzyme electrophoresis detected a circulating macroAST. M was discharged from the neonatal department at 72 hours of life. After 2 days was readmitted for weight loss and jaundice due to maternal hypogalactia, solved after rehydration and phototherapy. As in the mother, blood testing showed isolated AST elevation in the absence of clinical and biochemical signs of organ disfunction. Being aware of the maternal macroAST, M was not subjected to any procedure except for a liver ultrasound which was negative. The PEG precipitation study confirmed the presence of a macroAST even in the newborn. A follow-up evaluation at 2 months revealed a progressive decrease of AST activity in the infant’s serum, explained by the disappearance of macroAST of maternal origin (Table 1). The diagnosis of macroAST was added to the clinical file and the mother was reassured.

**Conclusion**

M showed an isolated AST elevation as a result of passively acquired maternal macroAST. Prompt diagnosis of macroAST let us to avoid unnecessary procedures in a neonate. To our knowledge, this is the first case of transplacental transfer of macroAST reported. Circulating macroenzymes should be suspected also in neonates whenever an isolated, unexplained increased enzyme activity is found, and the mother should be evaluated as source of that finding.

Informed consent to publish has been obtained from the parents.

**Table 1 (abstract O3).** Serum AST activity (U/L) in mother and daughter at birth (top) and two months later (bottom). Diagnostic cut-off for macroAST is a residual AST activity after PEG precipitation <30%

	Native sample	Sample after precipitation by PEG	% residual
**Mother**	762	36	**5%**
**Daughter**	940	62	**7%**
**Mother**	908	8	**1%**
**Daughter**	384	72	**19%**

## A16 Non primary Citomegalovirus infection in pregnancy: an underestimated problem

### Antonia Ruscitto^1^, Patrizia Macellaro^1^, Francesca Macchi^1^, Massimo De Paschale^2^, Claudia Pavia^2^, Maria Teresa Manco^2^, Pierangelo Clerici ^2^, Laura Pogliani^1^

#### ^1^ Department of Pediatrics, ASST Ovest Milanese, Legnano (Mi) ) (Italy); ^2^ Microbiology Unit, ASST Ovest Milanese, Legnano (Mi) ) (Italy)

##### **Correspondence:** Antonia Ruscitto (antonia.ruscitto@asst-ovestmi.it)

**Background**

Congenital Cytomegalovirus (cCMV) infection remains one of the major cause of hearing loss and neurodevelopment damage in developed countries, the neonatal treatment actually available has toxicity problems and limited efficacy. Congenital CMV infection can result not only from primary maternal infection but also from uncommon reactivation or re-infection. Therefore becomes important to individuate an universal neonatal screening to facilitate early detection and intervention.

**Case Report**

We describe two cases of cCMV infection caused by a virus reactivation or re-infection in immune women during first trimester of gestation and detected with Real-TimePCR on saliva samples. Case 1: C.S. female was born at term from CMV immune mother in first trimester. At 7 months of gestation her mother had a flu like syndrome. Neonatal course was uneventful.We founded two saliva samples positive for CMV DNA. Congenital infection was confirmed by urine test and maternal serologic exams. The newborn is actually symptomless. Case 2: C.F. male was born at 38+4 weeks of gestation from CMV immune mother in first trimester. At birth he showed axial hypotonia and mild jaundice. Also in this case two saliva samples resulted positive as blood and urine tests (data of newborns and mothers are shown in table 1and 2). Brain magnetic resonance imaging showed a subependimal hemorrhagic cyst and white matter alterations in both frontal cerebral hemispheres. Auditory Brainstem Response are in progress for suspected monolateral hearing loss. The two newborns were infected with CMV in utero in spite of maternal immunity before pregnancy. These infants are part of a bigger prospective study started on May 2019 in our department. Up to date 533 babies have been enrolled and in 3 of them (0,56 %) the congenital infection was diagnosed through saliva screening. It was possible to discriminate between primary infection (1 case) and nonprimary infection (2 described cases below) thanks to a retrospective analysis of serologic maternal data. The aim of our study will be to establish the prevalence of cCMV infection caused by a virus reactivation during pregnancy using saliva sample as diagnostic neonatal screening test.

**Conclusions**

Non primary cCMV infection is probably an underestimated problem**.** Universal neonatal screening for early detection of infected infants could improve early intervention for neurosensorial hearing loss and developmental delay specially for infants infected as a results of virus reactivation or re-infection in pregnancy.

Informed consent to publish has been obtained from the parents.

**Table 1 (abstract A16).** Case 1: Virological and serological data of mother and newborn

Case 1 mother					
Test	Gestational Age				
	3+2 weeks	9+4 weeks	17 weeks	30+5 weeks	post-partum
IgG	**POS** **(53 U/mL)**	**POS** **(60 U/mL)**	**POS** **(63 U/mL)**	**POS** **(69 U/mL)**	**POS** **(73 U/mL)**
Ig M	NEG (<5 .0 U/mL)	NEG (<5.0 U/ml)	NEG (<5.0 U/mL)	NEG (<5.0 U/mL)	NEG (5.68 U/mL)
IgG avidity	High (0.809)				
CMV-DNA serum	Undetectable	Undetectable	Undetectable	<593 cp/mL*	
CMV-DNA blood					Undetectable
CMV-DNA urine					Undetectable
Human milk					3776 cp/mL
Case 1 newborn					
Test	Age (days)				
	3	5	7		
IgG			POS (49.5 U/mL)		
IgM			POS (68 U/mL)		
1° saliva sample	4175374 cp/mL				
2° saliva sample		485949 cp/mL			
Urine		1572000 cp/mL			
Blood			3021 cp/mL		

* CMV-DNA detectable but below the quantization limit (593 cp/mL)

**Table 2 (abstract A16).** Case 2: Virological and serological data of mother and newborn

Case 2 mother				
Test	Gestational age			
	11+3 week	35+5 week	38+4 week	post partum
IgG	POS (159 U/mL)	POS (164 U/mL)	POS (162 U/mL)	POS (135 U/mL)
IgM	NEG (<5.0 U/mL)	NEG (<5.0 U/ml)	NEG (<5.0 U/mL)	NEG (<5.0 U/mL)
IgG avidity	High (0.774)			
CMV-DNA serum		Undetectable	Undetectable	
CMV-DNA blood				Undetectable
CMV-DNA urine				Undetectable
Case 2 newborn				
Test	Age (days)			
	3	6		
IgG		POS (133 U/mL)		
IgM		NEG (<5.0 U/mL)		
1° saliva sample	3769743 cp/mL			
2° saliva sample		6535042 cp/mL		
Urine		1144157 cp/mL		
Blood		32484 cp/mL		

## A17 Umbelical venous catheter (UVC) and pericardial tamponed in premature baby: a case report

### Federica Pontiggia, Roberto Bottino, Carmela Serlenga, Cristina Bellan

#### NICU and Neonatology, ASST-Bergamo Est, Seriate, Italy

##### **Correspondence:** Federica Pontiggia (federica.pontiggia@gmail.com)

**Background**

Cardiac tamponade is a rare, but often fatal, complication of central venous catheterization with an incidence of approximately 0.7%. [1, 2] Only its early recognition makes it possible to avoid cardiac arrest and death or severe neurological sequelae.

**Case report**

Male, 2nd twin, 29 week-old weighing 1300g, born by C-section after assisted medical procreation, with selective reduction of an embryo; IUGR and pPROM of the 1st twin. At birth positioned UVC, withdrawn after chest X-ray. On day 2, due to an increase in oxygen demand, a new X-ray performed shows a left pneumothorax (PNX). The baby is intubated and begins high-frequency oscillator ventilation (HFOV); a thoracic drainage is inserted with resolution of PNX. He undergoes several radiological examinations to check the pulmonary picture and since the UVC appeared to have migrated, his position is again optimized. On day 4 the baby, clinically stable with normal blood pressure without inotropic support and with negative infection indices, unespected shows tachycardia, with pale and marbled skin and with low arterial pressure, with conserved diuresis and normal saturimetry. To assess the need to proceed with filling or initiate inotropic therapy, an echocardiography is performed showing a pericardial effusion. Therefore we proceed to pericardiocentesis, with subxiphoid entry and we remove 9 mL of milky-white fluid with a clear improvement of the circle, raising of arterial pressure and reduction of the heart rate from 180 to 160 bpm. The UVC is removed, showing no signs of rupture or apparent malfunction, and a epicutaneous venous catheter (ECC) is positioned.

**Conclusion**

The etiology of cardiac tamponade is not entirely clear among the main hypotheses: direct lesion from the tip of the catheter or from its migration [1]; presence of areas of "weakness" due to incomplete muscularization of the myocardium associated with repeated catheter trauma [2]; from wall necrosis caused by infusion of hyperosmolar solution. [3, 4] In many cases this very serious complication occurs despite the apparent correct radiological position of the catheter. Probably a change in the methods of surveillance of the central catheters is necessary, as is currently proposed by the GAVeCeLT: ultrasound-guided positioning regarding umbilical accesses, positioning guided by the evaluation of the P wave with ECG trace, as regards the central catheters and ultrasound surveillance with the abandonment of X-ray, which has repeatedly proved to be less precise for this type of survey.

Informed consent to publish has been obtained from the parents.

**References**

1. Nowlen TT, Rosenthal GL, Johnson GL et al. *Pericardial effusion and tamponade in infants with central catheters.* Pediatrics. 2002; 110: 137-142

2. Arya SO, Hiremath GM, Okonkwo KC, Pettersen MD. *Central venous catheter-associated pericardial tamponed in a 6-day old: a case report.* Intern J of Pediatrics 2009; (6):910208

3. Sehgal A, Cook V, Dunn M. *Pericardial effusion associated, with an appropriately placed umbelical venous catheter.* J Perinatol. 2007; 27(5): 317-319

4. Mikako W, Thompson KS, Popek EJ et al. *Pericardial effusion and cardiac tamponade in neonates: sudden unexpected death associated with total parenteral nutrition via central venous catheterization.* Ann Clin Lab Sci. 2013; 43(2): 163-171

Written informed consent for publication of their clinical details was obtained from the parents of the patient. A copy of the consent form is available for review by the Editor of this journal

## A18 Post-traumatic subdural hygroma in newborns: a case report

### Alice Proto, Barbara Caruselli, Marco Fossati, Raffaele Masotina, Roberta Restelli, Stefano Martinelli

#### Division of Neonatology and Neonatal Intensive Care Unit – ASST Grande Ospedale Metropolitano Niguarda Ca' Granda, Milan, Italy

##### **Correspondence:** Alice Proto (alice.proto@ospedaleniguarda.it)

**Background**

Subdural effusion or subdural hygroma (SDG) is a common post-traumatic lesion following both major and minor head injury. [1] A trivial trauma can cause a separation of the dura arachnoid interface, which is the basic requirement for the development of SDG, with cerebral fluid storage in the subdural area. [2] Child's brain can be easily compressed and the skull is not fully developed. In trauma, it may compress brain tissue when impacted, causing coup rather than contrecoup injuries more commonly seen in adults. Acceleration-deceleration force causes the brain to move within the fixed venous channels and skull. Hemorrhages occur in the subarachnoid and subdural space if there is tearing of the superficial cortical veins. Most SDGs resolve spontaneously, but a few SDGs become chronic subdural hematoma (SDH) when the necessary conditions persist over several weeks. [3]

**Case report**

A 6 months old boy was admitted to our NICU after a car accident in which both parents were fatally injured. He was fastened to the car seat, conscious and crying, without any apparent traumatic lesions. The baby's left leg had a fracture across the tibia and fibula, which required a lower leg plaster cast. Cerebral TC scan and MRI showed subdural fronto-temporal-parietal bilateral effusion without cerebral median line shift and no skull bone fractures (Fig.1). Cerebral US performed over the next few days confirmed stability of subdural hygroma. However, on day 5, the infant showed sudden bilateral squint, an increase in head circumference and a bulging anterior fontanelle which required an external subdural drainage and a subsequent bilateral subdural peritoneal shunt placement. Subsequent brain MRI confirmed size reduction of subdural hygroma with and the baby was discharge after 32 days of stay. At discharge, his psychomotor evaluation didn't show any neurological impairment and oculistic symptoms vanished in few days after last surgical intervention.

**Conclusion**

Post-traumatic subdural fluid collections can produce increase in intracranial pressure and/or focal neurological deficits resulting from local compression on brain parenchyma. In these cases, the tear in the arachnoid acts as a ball-valve device [4] which prevents the restoration of cohesion within the dura arachnoid interface layer. [5] The goal of treatment of subdural fluid collections is to restore the cohesion within the dura-arachnoid interface layer [5]. Drainage of subdural fluid collections by means of subdural peritoneal shunt represents probably the most effective and safest treatment modality for chronic subdural fluid collections, as soon as any neurological symptoms appear.

Informed consent to publish has been obtained from the legal representative of the baby.

**References:**

1. Kumar R, Singhal N, Mahapatra AK. Traumatic subdural effusions in children following minor head injuriy. Childs Nerv Syst. 2008; 24:1391-1396

2. Lee KS. The pathogenesis and clinical significance of traumatic subdural hygroma. Brain Inj. 2008; 12:595–603

3. Murata K. Chronic subdural hematoma may be preceded by persistent traumatic subdural effusion. Neurol Med Chir (Tokyo).1993; 33:691–696

4. Oka H, Motomochi M, Suzuki Y, Ando K. Subdural hygroma after head injury. A review of 26 cases. Acta Neurochir (Wien).1972; 26: 265–273

5. M. Caldarelli, C. Di Rocco, and R. Romani. Surgical Treatment of Chronic Subdural Hygromas in Infants and Children. Acta Neurochir. 2002; 144: 581–588

**Fig. 1 (abstract A18).**
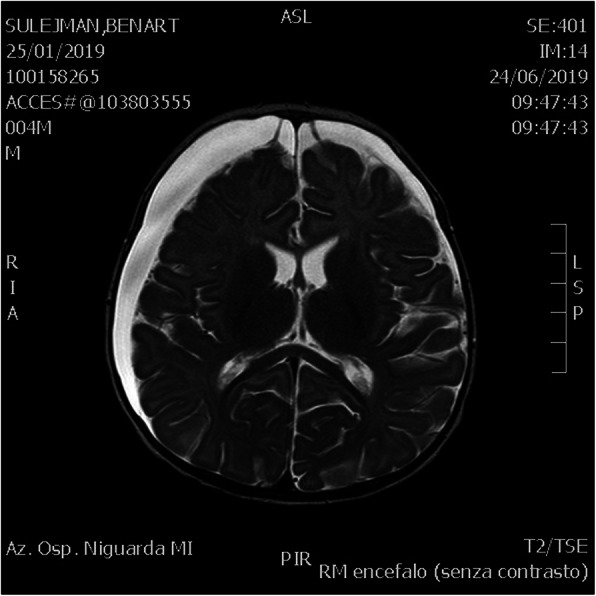
T2 RMN

## O4 Neonatal heart failure: viral myocarditis or Kawasaki disease?

### Alice Proto, Francesca De Rienzo, Gaia BM Chiesa, Italo Gatelli, Stefano Martinelli

#### Division of Neonatology and Neonatal Intensive Care Unit – ASST Grande Ospedale Metropolitano Niguarda Ca' Granda, Milan, Italy.

##### **Correspondence:** Alice Proto (alice.proto@ospedaleniguarda.it)

**Background**

Acute heart failure is a rare condition in the early neonatal period. Normally it is due to severe septicaemia, asphyxia or congenital heart malformations, but other causes must be considered. [1] Kawasaki disease (KD) is an auto-immune multiorgan vasculitis of still unknown origin with a predilection for coronary arteries and its diagnosis is based on the presence of ≥ 5 days of fever and four or more of the principal criteria including non-purulent conjunctivitis, non-specific skin rush, erythematous changes of the mucosae and of extremity and lymphadenopathy. However some signs or symptoms can be missing, especially in young children and newborns, leading to delay in diagnosis and high risk of complications. [2] Meanwhile, viral myocarditis can be due to a large spectrum of microrganisms, among which enterovirus are considered a leading cause. [3]

**Case report**

A 11 days-old male newborn was transferred to our NICU presenting severe heart failure requiring mechanical ventilation and inotrope support after a cardiac arrest. Parents referred fever in the past 5 days, conjunctivitis and lymphadenopathy. Echocardiography showed severe left ventricle disfunction with ejection fraction of 10%, severe left coronary artery dilatation (CA) and pericardic effusion. A gammaglobulins and i.v. therapy was promptly started and no response to high dose cathecolamine was observed. The steep deterioration of clinical condition, with multiple organ failure, and worsening of cardiac shock required artero-venous extracorporeal membrane oxygenation (ECMO) support, performed for 8 days with severe thromboembolic and cardiac complications leading up to newborn's death. Enteroviral polymerase chain reaction (PCR) tested on blood sample and myocardial biopsia resulted positive, but no genotyping is, at the moment, available.

**Conclusion**

Viral myocarditis and atypical KD are two entities that share many similarities and some clinical findings, like CA dilatations, can be present in both clinical scenarios. [4] While in KD artery dilatations are secondary to inflammatory vasculitis, in viral myocarditis the pathogenesis remains unclear even if vasodilatation due to fever and direct damage are considered potential mechanisms. [2] Therefore the diagnosis of an incomplete KD should be taken in to consideration not only in infants but also in newborn patients with heart failure. While the optimal treatment of viral myocarditis remains poorly studied [3], therapy with gammaglobulins can be crucial for the prognosis of KD and may prevent the development of aneurysms of the coronary arteries and improve the outcome significantly. [1]

Informed consent to publish has been obtained from the parents.

**References:**

1. Bolz D, Arbenz U, Fanconi S, Bauersfeld U. Myocarditis and coronary dilatation in the 1st weel of life: neonatal incomplete Kawasaki disease?. Eur J Pediatr. 1998; 157: 589-591

2. Rached-D'Astous S, Boukas I, Fournier A, Raboisson MJ, Dahdah N. Coronary artery dilatation in viral myocarditis mimics coronary artery findings in Kawasaki disease. Pediatr Cardiol. 2016 Aug;37(6):1148-5

3. Schlapbach LJ1, Ersch J, Balmer C, Prêtre R, Tomaske M, Caduff R, Morwood J, Provenzano S, Stocker C. Enteroviral myocarditis in neonates. J Paediatr Child Health. 2013 Sep; 49 (9): E451-4

4. Feldman AM, McNamara D. Myocarditis. N Engl J Med. 2000 Nov 9; 343(19):1388-98

## A19 Neonatal asphyxia and maternal carbon monoxide poisoning: which connection?

### Alice Proto, Alberto VR Brunelli, Marina Casartelli, Sofia Passera, Stefano Martinelli

#### Division of Neonatology and Neonatal Intensive Care Unit – ASST Grande Ospedale Metropolitano Niguarda Ca' Granda, Milan, Italy.

##### **Correspondence:** Alice Proto (alice.proto@ospedaleniguarda.it)

**Background**

Carbon monoxide (CO) exposure in pregnancy is a severe medical condition and the consequent fetal tissue hypoxia, mitochondrial disfunction and oxidative stress may have detrimental effect both on mother and fetus [1]. CO is a colorless and odorless gas formed by the partial burning of compounds like wood and fuel gases [2]. Affinity of CO to hemoglobin (Hb) is 200 times higher than oxygen (O_2_) causing tissue hypoxia; maternal CO crosses the placenta by passive diffusion and it combines with fetal hemoglobin, which is 3 times more affine to CO than adult Hb. [3, 4] Fetal outcome can be very variable depending on the severity of maternal involvement and the gestation age (GA). [5] Exposition during first trimester may lead to anatomical malformations and neurologic complication such as anoxic encephalopathy, especially involving periventricular white matter [6] while psychomotor and mental development may result from insult at any gestational age [4].

**Case report**

A 30-year-old, 40 GA, pregnant woman was admitted to our Emergency Dept due to domestic exposure to CO, complaining headache without loss of consciousness. The patient was immediately placed on 100% O_2_ and treated with Hyperbaric oxygen (HBO) therapy (2,5 ATA for 95 min) with a promptly decrease of COHb levels from 20% to 1,6%. After HBO cycle an emergent cesarean section was performed because of signs of fetal distress at the cardiotocography monitoring (CTG). At birth (male; 2,340 g; Apgar scores 3 at 1’ and 7 at 5’) cardiopulmonary resuscitation with endotracheal intubation was needed due to depressed breath drive and low oxygen saturation. A severe hypoxic ischemic encephalopathy was confirmed by electroencephalography and hypothermic treatment was performed, without any significant complications. During the first 12 hour of high dose O_2_ mechanical ventilation, COHb decreased from 6% to 1,4%. Cerebral magnetic resonance (MRI), performed on day 10^th^, showed multiple punctate frontal white matter lesions and venous infarctions in the right cerebellar hemisphere (Fig.1), still observable at the MRI performed 20 days after (Fig.2). The newborn was discharge on day 16th in good condition with planned neurological follow-up.

**Conclusion**

CO poisoning in pregnancy is an unusual but not rare cause in the context of HIE. [7] Despite relative safeness of HBO therapy in pregnancy, a CTG should be always performed to assess fetal wellness. After the stabilization of the mother, delivery should be planned in a tertiary level hospital. Neurological impairments must be considered and a neurological long-term follow-up must be planned also in asymptomatic newborns.[8,9]

Informed consent to publish has been obtained from the parents.

**References:**

1. Ernst A, Zibrak JD. Carbon monoxide poisoning. N Engl J Med. 1998; 339:1603-1608

2. Aubard Y, Magne I. Carbon monoxide poisoning in pregnancy. Br J Obstet Gynaecol. 2000; 107:833–838

3. Elkharrat D, Raphael JC, Korach JM et al. Acute carbon monoxide intoxication and hyperbaric oxygen in pregnancy. Intensive Care Med 1991; 7:289–292.

4. Culnan DM, Coffman BC. Carbon monoxide and cyanide poisoning in the burned pregnant patient: an indication for hyperbaric oxygen therapy. Ann Plast Surg 2018; 80:106-112

5. Yildiz H, Aldemir E, Altuncu E, Celik M, Kavuncuoglu S. A rare cause of perinatal asphyxia: maternal carbon monoxide poisoning. Arch Gynecol Obstet. 2010; Feb 281(2):251-4.

6. Delomenie M, Schneider F. Carbon monoxide poisoning during pregnancy: presentation of a rare severe case with fetal bladder complications. Case rep in Obstet Gynecol 2015; 687975.

7. Alehan F, Erol I, Onay OS. Cerebral palsy due to nonlethal maternal carbon monoxide intoxication. Birth Defects Res A Clin Mol Teratol. 2007 Aug;79(8):614-6.

8. Friedman P, Guo XM, Stiller RJ, Laifer SA. Carbon monoxide exposure during pregnancy. Obstet Gynecol Surv. 2015; Nov; 70(11):705-12.

9. Bothuyne-Queste E, Joriot S, Mathieu D, Mathieu-Nolf M, Favory R, Houfflin-Debarge V, Vaast P, Closset E, Subtil D. Ten practical issues concerning acute poisoning with carbon monoxide in pregnant women. J Gynecol Obstet Biol Reprod (Paris). 2014 Apr;43(4):281-7.

**Fig. 1(abstract A19).**
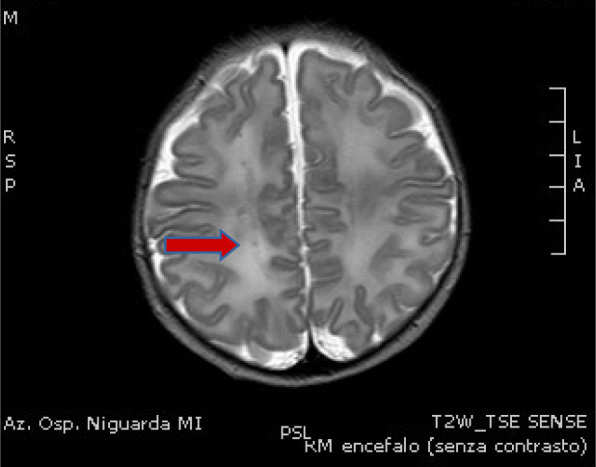
T2 RMN on day 10

**Fig.2 (abstract A19).**
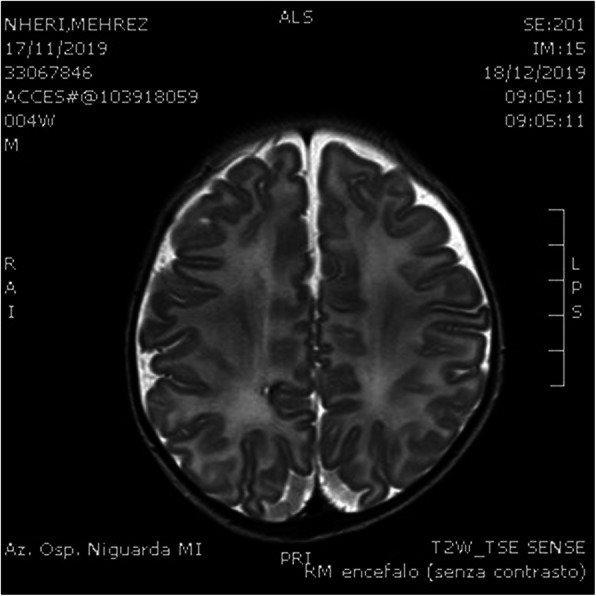
T2 RMN on day 30

## O5 Securing epicutaneo-caval catheters (ECC) in term and preterm neonates: a change of practice

### MariaGrazia Romitti MG, Carmen Rodriguez Perez, Elena Pezzotti

#### Neonatal Intensive Care Unit (NICU), Spedali Civili, Brescia, Italy

##### **Correspondence:** MariaGrazia Romitti MG (mg.romitti@alice.it)

**Background**

ECC is an essential device for neonates admitted to the NICU. Post insertion accurate management of this device is challenging but essential for the prevention of catheter-related complications. Proper securing of ECC prevents dislocation, micro-motions of catheter inside the vessel that causes “pistoning”, vein damage and eventually, thrombosis. Sealing the insertion site prevents bacterial contamination leading to infections. Blocking post insertion bleeding (Fig. 1A) allows to have a clean, visible and easy to monitor exit site (Fig. 1B). Cyanoacrylate sterile glue has proven to be effective for all the above topics.

**Materials and methods**

A careful review of literature concerning the securing of venous access and cyanoacrylate glue was made. The following data base have been searched: Medline, Embase, Emcare, Cochrane Library and Micromedex; copious were the findings about the use of cyanoacrylate for venous access; unfortunately nothing specifically related to neonates. From January to December 2018, all the exit sites of 95 ECC placed in neonates from 27 weeks of gestational age (Fig. 2), have been sealed with cyanoacrylate glue, covered with transparent tape. All exit sites were assessed, its score registered three times a day, dressing changed with insertion length checked weekly.

**Results**

95 exit sites of ECC sealed with cyanoacrylate have been assessed, 28 2fr and 67 1Fr catheters; score given was “zero” (no inflammation) to all, insertion length was checked weekly during change of dressing and found unmodified.

**Conclusions**

The sterile glue stabilizes the venous catheter, therefore preventing its movements and micro-motions, reducing pistoning. It provides a secure fixation against dislodgements (Fig. 1C), it makes it easier and less risky to change the dressing. The cyanoacrylate glue has proven to have haemostatic properties and antibacterial effects. It does make assessing and dressing easier, faster and safer with less products utilized (gauzes, strips, haemostatics). The cyanoacrylate sterile glue is now fully in use in our unit for the securing all ECC exit-sites and has proved to be effective, safe, feasible, easy to introduce and keep, cost and time saving.

**Fig. 1 (abstract O5).**
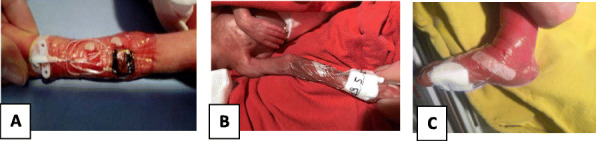
Comparison between previous (A) and new procedures to stop post insertion bleeding (B) and dislodgement (C)

**Fig. 2 (abstract O5).**
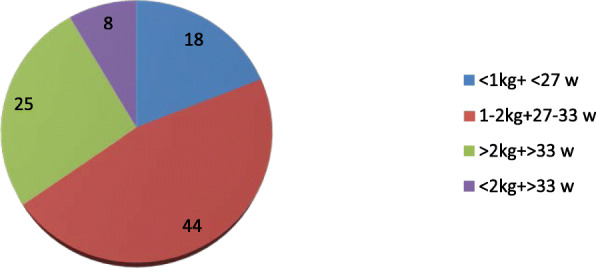
Distribution of neonates according to weight and gestational age

## A20 Floppy infant: one diagnosis is not enough

### Giulia Russo, Maddalena Bove, Carmen Bucolo, Patrizia Corsin, Gisella Garbetta, Laura Lorioli, Giulia Tronconi, Rosanna Rovelli, Antonella Poloniato, Graziano Barera

#### U.O. di Neonatologia e Patologia neonatale - IRCCS Osp. San Raffaele, Università Vita-Salute San Raffaele, Milano, Italy

##### **Correspondence:** Giulia Russo (russo.giulia@hsr.it)

**Background**

Neonatal hypotonia is a frequent finding at the first examination of the newborn. Finding the real cause of hypotonia often requires a much more complex diagnostic process, since various disorders can underlie this condition.

**Case report**

D.T. is born at term, small for gestational age; no family history of hereditary disease. The pregnancy was complicated by IUGR and gestational hypothyroidism. Apgar 7/9. At birth hypotonia, ligamentous laxity, facial dysmorphisms (hypertelorism, micrognathia), cryptorchidism and weak crying were evidenced. As diagnostic workup, to exclude any cause of central hypotonia, EEG, ophthalmological examination, brain MRI, acoustic and visual evoked potentials were performed and resulted within limits. At blood tests muscle or liver necrosis enzyme resulted within normal limits, as well as metabolic tests. Thus, a wide genetic workup was performed. CGH-array was performed and documented a microdeletion of the distal portion of the short arm of chromosome 5 (5p15.33). This alteration is included in the chromosomal region associated with the Cri-du-chat syndrome, but does not involve the region that is identified as responsible for the clinical sign of the "cri du chat", while contributing in determination of the phenotype of patients with 5p partial monosomy (as variable-degree psychomotor retardation, language delay, dysmorphic notes, hypotonia and cryptorchidism). Methylation analysis of chromosome 15 (15q11-13) was also performed, highlighting the absence of the paternal allele as from uniparental disomy or imprinting defects, compatible with the diagnosis of Prader Willi syndrome (PWS).

**Conclusion**

PWS is one of the main diseases involved in the differential diagnosis of floppy infant. In 65-70% of cases the PWS results from a deletion of the 15q11.2-q13 region on the chromosome of paternal origin; in 20-30% of cases PWS is caused by maternal uniparental disomy for chromosome 15, and in 1-3% by imprinting defects (where diagnosis is possible only by methylation test). Cri du Chat syndrome is a chromosomal disease due to the deletion of a variable portion (5-40 Mb) of the short arm of chromosome 5 (5p-). The peculiarity of our case lies in the coexistence of two rare genetic syndromes in a hypotonic newborn whose neuromotor and phenotypic features were suggestive for the Prader Willi syndrome. The diagnosis of Cri-Du-Chat syndrome was unexpected because of the absence of the characteristic clinical sign. In this case, the combined use of two different genetic investigation techniques (CGH array and methylation test) was fundamental to allow the diagnosis.

Informed consent to publish has been obtained from the parents.

## A21 Never underestimate an isolated hypocalcemia

### Giulia Russo, Valentina Biffi, Gilda Cassano, Dario Gallo, Laura Lorioli, Benedetta Mariani, Gaia Vincenzi, Antonella Poloniato, Rosanna Rovelli, Graziano Barera

#### U.O. di Neonatologia e Patologia neonatale- IRCCS Osp. San Raffaele, Università Vita-Salute San Raffaele, Milano, Italy

##### **Correspondence:** Giulia Russo (russo.giulia@hsr.it)

**Background**

Hypocalcemia is a common metabolic problem in newborn period. Hypocalcemia is defined as early-onset, if it occurs within the first 72 h of life, or late-onset, which is usually symptomatic and occurs after the first 72 h. Early-onset hypocalcemia is more common in preterm infants, IUGR infants, infants with perinatal asphyxia or born of a diabetic mother. The most common causes of late-onset hypocalcemia include hypomagnesemia, hypoparathyroidism, excessive phosphate intake and vitamin D deficiency.

**Case report**

S.R. is born at 38 + 3 GW from vaginal delivery, third-born child, no family history of hereditary disease. Apgar 9/10 (1^st^ and 5th minute). Birth weight 3490 g. At first evaluation we evidenced facial dysmorphisms, as narrow and tapered palpebral rhymes, roundish face, micrognathia, slightly low ear implantation, mild hypertelorism. Heart ultrasound, brain ultrasound, ophthalmological examination, acoustic and visual evoked potentials resulted within limits. The brain MRI showed a large sylvian cistern and a slightly simplified aspect of cerebral convolutions, particularly in the frontal area. At the abdomen ultrasound mild dilation of left calico-pyelic cavities was evidenced; renal function tests and standard urine tests within limits. On the 5th day of life, blood tests showed asymptomatic hypocalcemia, hypomagnesemia, hypovitaminosis D and hypoparathyroidism. Interstingly, total blood count and white blood cells subpopulation were within normal range. Maternal blood tests resulted within limits. Therefore, therapy with calcium gluconate, vitamin D and magnesium sulfate intravenously was started, with subsequent correction of dyselectrolytemia. Considering the electrolyte unbalance and the phenotypic characteristics, we performed array-CGH analysis, that showed the presence of a microdeletion on the long arm of a chromosome 22 (22q11.21), which involves the chromosomal region associated with DiGeorge syndrome/Velo-cardio-facial syndrome (VCFS).

**Conclusion**

The 22q11.1 microdeletion syndrome has an estimated incidence of 1:1000/1:4000 live births. Characteristic signs and symptoms may include birth defects such as congenital heart disease, palate abnormalities, immunological and autoimmune disorders, neonatal late-onset hypocalcemia, renal malformations (such as hydroureteronephrosis), genital anomalies, CNS malformations (polymicrogyria), gastrointestinal malformations, skeletal involvement, facial anomalies and recurrent infections, due to problems with the immune system's T cell-mediated response. In our case the association of late onset hypocalcemia and dysmorphic aspects raised the suspicion of 22q11.1 deletion syndrome, which was then confirmed by the execution of the genetic analysis using CGH-array.

Informed consent to publish has been obtained from the parents.

## A22 Stroke neonatal in susptected neonatal rendu Osler Syndrome: description of a clinical case

### Elena Sala, Anna Tulone, Isabella Formica, Stefania Ferrari, Giovanna Mangili

#### Neonatal Intensive Care Unit, ASST PG XXIII, Bergamo (Italy)

##### **Correspondence:** Elena Sala (elena-sala@libero.it)

**Background**

Hereditary Hemorrhagic Telangiectasia (HHT) is an angiogenetic disease causing arteriovenous dilations: mucocutaneous telangiectases and visceral shunts. The prevalence of HHT is approximately 1-5/10.000. Patients affected by HHT probably have a reduced life expectancy, but it’s highly dependent by the presence of visceral complications. The onset of symptoms in neonatal period is quite rare and can be associated with severe clinical manifestations.

**Case report**

Leonardo was born in another Centre, by eutocic birth, at term (39,4 w) and AGA. Perinatal period was normal. At 25 days Leonardo presents clonus of the left hemisome, so he is admitted to PS of other Hospital and then transferred to our NICU. From the anamnestic review at our Centre, a close familiarity to HHT emerges (father, paternal grandmother and uncle affected). During the hospital stay Leonardo didn’t manifest any further critical events. The electroencephalographic study showed a normal pattern. The neuroradiological tests performed (brain MRI and brain ultrasound) documented the presence of subacute hemorrhagic lesions in the left cerebellar area (point), right rear thalamic area (8mm) and in the right rear frontal area (Rolandic area), the latter larger (2x1 cm). These findings, together with the familiarity for HHT, have suggested that Leonardo is affected by HHT too (clinical diagnosis probable according to the *Curaҫao diagnostic criteria)* and that hemorrhagic manifestation involved areas of abnormal vascularization. Abdomen ultrasound was performed for screening and it excluded visceral MAV. The NPI pre-discharge assessment was normal. Genetic testing for the diagnostic definition of certainty is still ongoing. Hemorrhagic lesions re-evaluated by MRI, about a month after the acute event, appeared in regression. Now the patient carry on a multidisciplinar follow-up in another Centre; he repeated abdomen ultrasound and cardiological evaluation that remaine negative.

**Conclusion**

Rendu-Osler-Weber disease is an autosomal dominant disorder that mainly involves blood vessels. The most frequent symptomatic triad consists of chronic epistaxis, iron deficiency anemia and muco-cutaneous telangiectases that increase in number with age. The expression of the disease is very variable because visceral arterovenous malformations (MAVs) can be asymptomatic or cause complications. Mutations in ACRVL, ENG and, more rarely, SMAD4 genes causing the disease. The follow-up of these patients is aimed at identifying MAVs and at their possible treatment with occlusion by interventional radiology.

Informed consent to publish has been obtained from the parents.

## A23 “DIAGNOSTIC ODISSEY IN NEONATAL EPILEPSY”: WHEN THE EXOME?

### Felicia Varsalone^1^, Renzo Guerrini^2^, Roberta Maffioli^1^, Maurizio Felice^1^, Cristina Bellan^1^

#### ^1^U.O. Neonatologia – T.I.N . ASST Bergamo-est “Bolognini” Seriate (Bg) ) (Italy); ^2^U.O. Azienda Ospedaliero-Universitaria “A.Meyer” Clinica di Neurologia Pediatrica- Università degli Studi di Firenze ) (Italy)

##### **Corresponding author:** Felicia Varsalone (felicia.varsalone@asst-bergamoest.it)

**Background**

The gene CACNA1G located on the long arm of chromosome 17, encodes for calcium dependent voltage channels that mediates the entry of calcium ions into excitable cells (highly expressed in Purkinje neurons and deep cerebellar nuclei). Mutations in heterozygous of this gene are associated with Spinocerebellar ataxia and early onset Spinocerebellar ataxia with neurodevelopmental deficit, all transmitted in autosomal dominant mode [1].

**Case report**

Born at term. Outborn, unrelated parents. AGA. Obstetric history: Pregnant cholestasis. Pathological history: in fist day respiratory distress resolved after administration of oxygen in the incubator (FiO2 0,30). Family history: familiarity for epilepsy (paternal cousin). Discharged on the fourth day of life in good general condition. At 17 days of life onset of hypertension, difficult sucking, poor reactivity, perioral cyanosis and deviation of gaze and subsequent seizures characterized by incoming hypertension, cyanosis, automatic sucking for which treatment with phenobarbital was undertaken with remission of clinical symptoms while persisting critical electrical activity. Admission to our NICU. Objective examination: palmate fingers 2°-3° right hand and 4° - 5° left hand. Normal: infectious, metabolic, liquorculture, karyotype, CGH-Array, genetic panel for epilepsy and instrumental examinations (brain ultrasound and MRI). The EEG: "asymmetry of electrical activity, anomalies of specific significance on both hemispheres". The analysis of the exoma (Laboratory of Neurogenetics of the AOU "A.Meyer"-Florence) showed a variant heterozygous de novo (C4591A>G/p.Met1531Val) in the gene CACNA1G. Currently our patient is 14 months old, has a serious neuromotor and cognitive delay and complete remission of critical episodes.

**Conclusion**

In the literature four patients with de novo CACNA1G mutations are reported, severe motor and cognitive disabilities, cerebellar atrophy, facial dysmorphisms, digital abnormalities, microcephaly and epilepsy are described [2]. Two of the four cases described have the same phenotype and genetic variant of our patient [3]. It is estimated that 42% of developmental disorders are caused by de novo mutations, in selected cases continuing genetic investigations with the study of the exome may lead to identify de novo mutations that could be pathogenic pathologies not yet described.

Written informed consent for pubblication of their clinical details was obtained from the parents of the patient. A copy of the consent form is available for review by the Editor of this journal

**References:**

1. Coutelier M., Blesneac I. A Recurrent Mutation in CACNA1G Alters Cav3.1 T-Type Calcium-Channel Conduction and Causes Autosomal-Dominant Cerebellar Ataxia Am J Hum Genet. Am J 2015 Nov 5;97(5):726-37

2. Scocchia A., Wigby K.M. Clinical whole genome sequencing as a first-tier test at a resource-limited dysmorphology clinic in Mexico. NPJ Genom Med. 2019 Feb 14;4:5.

3. Chemin J., Siquier-Pernet K. De novomutation screening in childhood-onset cerebellar atrophy identifies gain-of-function mutations in the CACNA1G calcium channel gene. Brain. 2018 Jul 1;141(7):1998-2013.

## A24 The importance of recognizing Dumping Syndrome after Nissen fundoplication in children

### Laura Pagani^1^, Aurelia Castiglione^1^, Luciana Leva^1^, Elisabetta Villa^1^, Claudia Maria Pagliotta^1^, Paolo Bini^1^, Mario Barbarini^1^

#### ^1^Neonatal Intensive Care Unit, ASST Lariana, San Fermo della battaglia, Como, Italy

##### **Correspondence:** Laura Pagani (laura.pagani@asst-lariana.it)

**Background**

Dumping syndrome is infrequently reported, but known to occur after Nissen fundoplication in children.

**Case report**

The patient was born at 37 weeks of gestation from mother with diabetes poorly controlled. In delivery room she needed mask ventilation with benefit (Apgar score 4/9 and umbelical cord pH 6,95, BE -13,7, lactate 11,2). At one hour of life she suffered of severe hypoglycemia treated with glucose infusion (max glucose rate 13 g/kg/die) for 5 days. Since the second week of life she presented poor suction and recurrent vomiting little responsive to thickening of the formula and antiacid medication. The diagnosis of severe gastroesophageal reflux was made by radiological investigations. At first a transpyloric tube was placed, then at 5 months of life the baby underwent Nissen fundoplication. After a few days, enteral nutrition via gastric tube was started. Vomiting, profuse sweating and restlessness occurred in each postprandial period. These syntoms were associated with hypoglycemia or hyperglycemia. Continuous glucose monitoring was performed using a sensor inserted into subcutaneous tissue. The monitoring revealed remarkable postprandial glucose rises and subsequent sharp declines. These findings were suggestive for Dumping Syndrome. Continuous nutrition was started with success; later Acarbose, a glucosidase inhibitor that slows carbohydrate digestion, was started in addition to nutrition via percutaneous endoscopic gastrostomy (first formula milk + maizena, then also meal) for about one month, then it was gained a good glicemic status just with diet.

**Conclusion**

Dumping syndrome is a frequent complication in infants after Nissen fundoplication (around 30% of cases). However, it is sometimes difficult to diagnose in infants, because symptoms may be subtle and non‐specific and the glycemic change may be overlooked by a limited frequency of blood sampling. The syndrome is characterized by an early phase (30-60’ after the meal) of hyperglycemia associated with abdominal distension, vomiting, diarrhea, tachycardia, irritability, followed by a late phase (1-2 hours later) characterized by a reactive severe hypoglycemia. This second phase often asyntomatic, could be dangerous. It is believed that precipitous emptying of carbohydrate containing solutions from the stomach into the upper small bowel and subsequent hyperglycemia stimulate excessive insulin secretion bringing to severe hypoglycemia. Continuous glucose subcutaneous monitoring is safe and tolerable both in neonates and infants and so it could be a good tool for identify dumping syndrome after Nissen fundoplication.

Informed consent to publish has been obtained from patient’s parents.

## A25 A rare case of Usher syndrome

### Laura Pagani, Roberta Barachetti, Gaia Natalé, Gabriele Rulfi, Anna Pagliaro, Aurelia Castiglione, Elisabetta Villa, Mario Barbarini

#### Neonatal Intensive Care Unit, ASST Lariana, San Fermo della battaglia, Como, Italy

##### **Correspondence:** Laura Pagani (laura.pagani@asst-lariana.it)

**Background**

Usher syndrome is a disorder characterized by retinitis pigmentosa and congenital sensorineural hearing loss. It is a rare disorder with an incidence of 3 to 4,4 per 100,000 people.

**Case report**

The proband is the second child of non relative healthy Italian parents born to 38+6 weeks pregnancy and uncomplicated vaginal delivery with Apgar score 7-9 at 1 and 5 minute respectively. His birth weight was 3,680 Kg (75-90°C). Prenatal ultrasound showed renal bilateral pielectasia without oligohydramnios. The kidney US , at birth, revealed bilateral hydronephrosis with hydroureters and bladder hypertrophy as it tries to overcome the outflow obstruction. Therefore a bladder catheter was inserted. Serum creatinine was 2,53 mg/dl , urine culture negative with high level of PCR (78,8 mg/L). Then he started an antibiotic therapy. In suspected PUV (Posterior urethral valves) a Micturating Cystourethrogram (MCU) confirmed a wide bladder with multiple diverticulae, dilated posterior urethra and severe bilateral vesicouretheral reflux. The patient underwent to PUV fulguration after a sovrapubic cystostomy with remission of the renal failure and improvement of symptoms a nd signs of obstructive syndrome. In addition the routine hearing test and the audiometry have showed a profound hearing loss. There was no evidence of other congenital malformation, no ocular anomalies. Citomegalovirus infection excluded. After acquisition of informed consent by the parents, the genetic test EXOME was performed. The test detected two mutations in heterozygosity c.7482+1G>A and c.9510+5G>A of CDH23 gene. These mutations could be linked to the Usher Syndrome.

**Conclusion**

Usher syndrome is the most common cause of combined hearing and visual loss and is transmitted through an autosomal recessive inheritance. The scientific literature are still not confirmed the association with urinary tract malformation. We will follow-up the baby to look for ocular abnormalities.

Informed consent to publish has been obtained from patient’s parents.

## A26 Segmental overgrowth syndromes: clinical criteria to guide diagnosis in a male newborn

### Raffaella De Santis^1^, Laura Gianolio^1^, Francesco Cavigioli^1^, Irene Daniele^1^, Petrina Bastrenta^1^, Enrica Lupo^1^, Francesca Castoldi^1^, Silvia Bianchi^1^, Andrea Righini^2^, Enrico Alfei^3^, Luigina Spaccini^4^, Gian Vincenzo Zuccotti^1^ and Gianluca Lista^1^

#### ^1^Department of Pediatrics-NICU Ospedale dei Bambini “V. Buzzi”, ASST-FBF-Sacco, Milan, Italy; ^2^Department of Radiology Ospedale dei Bambini “V. Buzzi”, ASST-FBF-Sacco, Milan, Italy; ^3^Department of Neurology Ospedale dei Bambini “V. Buzzi”, ASST-FBF-Sacco, Milan, Italy; ^4^Department of Genetics Ospedale dei Bambini “V. Buzzi”, ASST-FBF-Sacco, Milan, Italy

##### **Correspondence:** Raffaella De Santis (raffaella.desantis@unimi.it)

**Background**

MCAP (Megalencephaly-capillary malformation) is a rare condition in the heterogeneous group of segmental overgrowth syndromes; these are caused by mosaic somatic mutations in genes involved in cellular growth, such as PI3KCA. Less than 200 cases are reported in literature, probably because frequently misdiagnosed. Over the last decades, there have been several attempts to define possible diagnostic criteria and the syndrome has often been renamed. The latest definition [1] emphasizes the two major findings (M-egalencephaly and cutaneous CAP-illary malformations) and classifies all the clinical features into 5 main groups (Table 1).

We report a case satisfying these recent clinical criteria.

**Case report**

M., male, born at 39 weeks’ GA via cesarean section. BW 4550 g, length 53 cm and occipitofrontal circumference (OFC) 39 cm (all parameters > 97° percentile). Perinatal course: only few episodes of asymptomatic hypoglycemia. A widely open anterior fontanelle and right-side dominant limb hemihypertrophy were observed. Multiple net-like capillary malformations were found on trunk and limbs (Fig. 1). A slightly decreased muscle tone was observed at neurological evaluation; EEG: relatively well-organized activity with rare spikes and slow-wave bursts; brain MRI: dysmorphic sylvian fissures associated to a polymicrogyria-like cortical malformation mainly involving frontal lobes (Fig. 2).

The cardiological evaluation showed a moderately discrepant diameter between ascending and transverse aortic arch with no blood flow impairment. No further anomalies were observed at ophthalmologic examination and abdominal ultrasonography was normal. After hospital discharge no evident neurological impairments have been detected so far and capillary malformations gradually became lighter. However, a brain ultrasonography performed at age one, due to a progressive increase of OFC, showed a drastic lateral ventricles’ enlargement; a second brain MRI is going to be performed soon.

**Conclusion**

A clinical diagnosis of MCAP could be formulated in our case. A genetic confirmation through a molecular analysis of genes involved in PI3k/AKT/mTOR pathway is ongoing in order to correlate MCAP phenotype with the causative genotype. Moreover, a strict surveillance of brain anomalies and neurological development is required to evaluate possible future impairments.

Informed consent to publish has been obtained from this patient.

**Reference**

1. Mirzaa GM, Conway RL; Megalencephaly-capillary malformation (MCAP) and megalencephaly-polydactyly-polymicrogyria-hydrocephalus (MPPH) syndromes: two closely related disorders of brain overgrowth and abnormal brain and body morphogenesis.,» *Am J Med Genet A,* vol. 158A(2), pp. 269-291, 2012 Feb.

**Fig. 1 (abstract A26).**
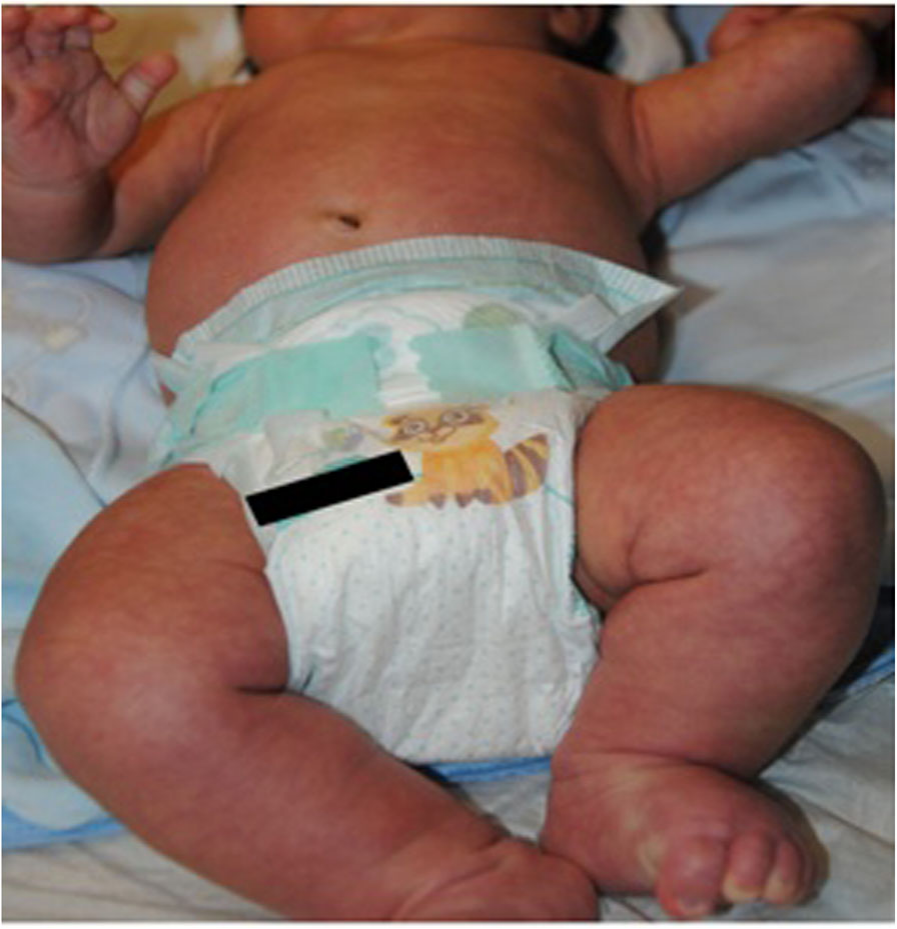
Limb asymmetry and capillary malformations

**Fig. 2 (abstract A26).**
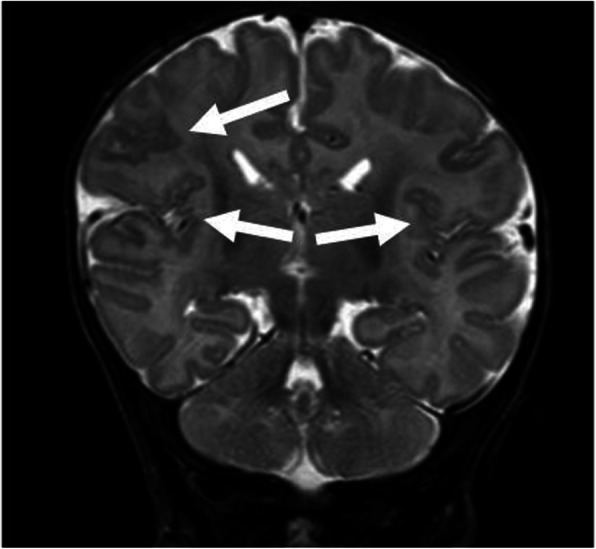
Polymicrogyria at brain MRI (T2-weigthed)

**Table1 (abstract A26).** Diagnostic Criteria for MCAP: core feature (1) plus either (2) or (3)

Core Features	Supportive Features	Secondary Features
**(1) Early overgrowth (brain > somatic tissue) progressive megalencephaly**	Selective brain overgrowth (e.g. ventriculomegaly); Congenital somatic overgrowth Somatic or cranial asymmetry	Hypotonia Frontal blossing or dolichocephaly Seizures Developmental delay
**(2) Developmental vascular disorders capillary malformations (midline face or body widespread)**	Infantile hemangiomas, venous aneurysms, aberrant vasculature	
**(3) Distal limb anomalies (syndactily)**	Polydactyly, Sandal-gap toes	
**(4) Cortical brain malformations (polymicrogyria)**		Seizures Developmental delay
**(5) Connective tissue dysplasia**		Hypotonia

## A27 Proceedings of Neonatal Clinical Lung Ultrasound in meconium aspiration syndrome: a new diagnostic frontier that’s becaming reality

### Alexandre Diouf, Giuseppe Limoli, Caterina Sabatini, Roberta Giacchero

#### Department of Pediatrics and Neonatology, Ospedale Maggiore di Lodi, ASST Lodi, Italy

##### **Correspondence:** Alexandre Diouf (diouf.sedar@gmail.com)

**Background**

Meconium aspiration syndrome (MAS) is a common cause of severe respiratory distress in neonates, due to a life-threatening lung injury induced by meconium in the airways. Early diagnosis and treatment are important for improving the prognosis. Historically, MAS has been mainly diagnosed based on medical history, clinical manifestations, and chest X-ray. In recent years ultrasonography has been used successfully to diagnose many types of lung diseases, such as pneumonia, pleural effusion, respiratory distress syndrome, transient tachypnoea of the newborn. Lung ultrasound (LUS) can be performed at the bedside, provides accurate diagnostic information when compared with CT scans and chest radiographs, and it has the additional benefit of being non-irradiating and easily repeatable with no side effects for the patient. LUS is increasingly being used in pediatric and neonatal critical care settings and specific international recommendations for bedside use have already been elaborated. Recently LUS findings have been formally described and confirmed in a larger study of neonates with MAS. We report a peculiar neonatal clinical case with early LUS diagnosis of MAS.

**Case report**

The patient was born full-term by emergency caesarean section, due to signs of fetal distress. At birth endotracheal suction was carried-out due to non –vigorous new-born with evidence of meconium- stained amniotic fluid , followed by ventilation with T-piece resuscitator. After stabilization in delivery-room the new-born was admitted to neonatal intensive care unit (NICU) and non invasive respiratory support was continued through nasal C-PAP. LUS was early performed with evidence of pleural line anomalies, atelectasis and alveolar-interstitial syndrome or B-line in the non-consolidation area. Clinical and ultrasound findings was strongly suggestive for MAS. Point of care cardiac ultrasound was then carried out with evidence of high pulmonary pressure and patent ductus arteriosus with bidirectional shunt. The patient after an early bedside and complete diagnosis was then transferred to the NICU of our Hub third-level center for subsequent advanced management.

**Conclusion**

Clinical LUS is a simple, useful, reliable, accurate, bedside and “radiation-free” technique for the diagnosis of the main neonatal lung diseases. LUS findings have been formally described and confirmed in a larger study of neonates with MAS. As with a lot of other lung disease, time has now been taken to consider lung ultrasonography as the main diagnostic tool also for MAS.

Informed consent to publish has been obtained from this patient.

## A28 Evocative coronary artery aneurisms in incomplete Kawasaki disease in a 12-weeks old infant effectively managed with anakinra

### Alessandra Maggio^1^, Federico Fortuni^2^, Irene Ioimo^1^, Michela Coccia^2^, Chiara Gagliardone^3^, Alessia C. Codazzi^3^

#### ^1^University of Pavia, Department of Pediatric, IRCCS Policlinico San Matteo. (Italy); ^2^University of Pavia, Department of Cardiology, IRCCS Policlinico San Matteo. (Italy); ^3^Department of Pediatric Cardiology, IRCCS Policlinico San Matteo. (Italy)

##### **Correspondence:** Alessandra Maggio (m.alessandra_a@libero.it)

**Background**

Kawasaki disease (KD) is an inflammatory vasculitis of unknown origin and the most common cause of acquired cardiac disease in children in developed countries. Coronary artery aneurysms (CAA) develop in 20-25% of untreated or delayed treated patients. In this case report we present an evocative case of incomplete KD complicated by coronary aneurisms treated with Anakinra.

**Case report**

A 2-month-old boy was admitted to the pediatric department for fever and after 2 days, for worsening of clinical conditions, was transferred to Neonatal Unit. He showed remittent fever lasting for a week associated with conjunctivitis, diffuse skin rash and cheilitis. The laboratory tests showed increased inflammatory indexes, hypertransaminasemia, hypoproteinemia and high platelet count: an incomplete KD was diagnosed. According to the guidelines of the Italian Society of Pediatrics high-dose intravenous Immunoglobulin were administered two times; and acetylsalicylic acid (ASA) and methylprednisolone were started. After 10 days an echocardiogram was performed and showed the presence of an increased echogenicity at the level of the proximal right coronary artery (RCA). 5 days later the inflammatory indexes increased again and the fever reappeared, a new echocardiogram showed the presence of two saccular aneurisms at the proximal RCA (first diameter: 2.8 mm [Z-score 5.15]; second diameter: 2.7 mm [Z-score 4.80] – Fig. 1A) and the left main artery (LMA) appeared hyperechogenic and slightly ectasic (2.8 mm [Z-score: 3.05]- Fig. 1B). Fig. 1A Fig. 1B

To avoid potentially fatal complications related to the aneurisms a dual antiplatelet therapy with clopidogrel and ASA was started. A total body CT scan excluded the presence of other aneurisms. The clinical, laboratory and imaging features were suggestive for high risk of progression of the coronary aneurisms and therefore Anakinra was administered. The clinical conditions and laboratory tests steadily improved without any relapse. After 9 weeks of Anakinra the echocardiograms showed a complete regression of the sings of inflammation at the coronary arteries without any aneurism.

**Conclusion**

This case underlines the importance to promptly diagnose KD in order to set up the right treatment and avoid complications. Moreover, it stresses the importance of following up these patients with echocardiograms to identify progression of the disease and to avoid acute complications and further sequelae in the future related to the presence of CAA. Even if the optimal treatment for IVIG non-responsive patients remains controversial and there are few dates about interleukin-1 receptor antagonists, Anakinra proved to be an effective treatment.

Informed consent to publish has been obtained from this patient.

**Fig. 1 (abstract A28).**
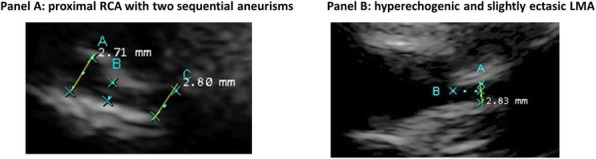
See text for description

## A29 An insidious “gut killer” in Extremely Low Birth Weight Infants

### Laura Morlacchi^1^, Silvia Pisoni^1^, Mirella Mogiatti^2^, Angela Bossi^1^, Valerio Gentilino^2^, Massimo Agosti^1^

#### ^1^NICU - Woman and Child Department, F. Del Ponte Hospital, Varese, Italy; ^2^Pediatric Surgery Unit - Woman and Child Department, F. Del Ponte Hospital, Varese, Italy.

##### **Correspondence:** Laura Morlacchi (laura.morlacchi@asst-settelaghi.it)

**Background**

Spontaneous intestinal perforation (SIP) is a specific medical condition commonly occurring in Extremely Low Birth Weight (ELBW) infants. Early use of postnatal steroids and indomethacin and some pathogens (Coagulase-Negative Staphylococci and Candida species) could play a key role in its etiology. [1]

**Case reports**

C1 is a preterm male infant (gestational age-GA 24 weeks, 540 g), born after caesarean section, premature rupture of membranes from the 16^th^ week. Intubated in delivery room, Apgar 4/4/6. Antibiotic therapy (ampicillin and gentamicin) and fungal prophylaxis with fluconazole were immediately started. In the first day of life he developed a hypertensive pneumothorax successfully treated with chest drainage. Trophic diet with fresh human milk was initiated on day 1. From day 2, patent ductus arteriosus pharmacological treatment with ibuprofen and acetaminophen was started, without success. On day 7 after birth the infant presented clinical worsening (abdominal distension and discoloration, leucocytosis) and evidence of pneumoperitoneum on the X-ray. A peritoneal drainage was inserted; after 3 days, due to failed clinical improvement, the patient underwent urgent laparotomy, revealing a jejunal focal perforation (figure 1). Blood, urine and peritoneal fluid cultures were positive for Candida Krusei (CK). Liposomal amphotericin B was administered for 21 days.

C2 is a preterm male infant (GA 24+5 weeks, 750 g), born after eutocic delivery, maternal vaginal swab positive for CK. Intubated in delivery room. Apgar 2/4/7. Antibiotic therapy (ampicillin and gentamicin) and prophylactic fluconazole were immediately started. Trophic diet with fresh human milk was initiated on day 1. He underwent spontaneous closure of ductus arteriosus. On day 7 after birth, he showed clinical deterioration (abdominal distension, leucocytosis and hyperlactacidemia). Radiological findings revealed pneumoperitoneum (figure 2a and figure 2b). Peritoneal drainage was successfully maintained for thirteen days. CK was found in blood, urine and peritoneal fluid cultures. Liposomal amphotericin B was administered for 25 days.

**Conclusion**

About 30% of SIP is associated with systemic candidiasis [2]. The incidence of CK in ELBW is estimated at around 0.2% of all candidemia. Intestinal involvement after systemic candidiasis is a dangerous complication. Due to its intrinsic fluconazole resistance, CK has been recognised as a potentially multidrug resistant pathogen. In all neonates with SIP a peritoneal fluid culture is mandatory to direct the most effective antimicrobial treatment.

Informed consent to publish has been obtained from this patient.

**References**

1. Gordon PV. Understanding intestinal vulnerability to perforation in the extremely low birth weight infant. Pediatr Res. 2009 Feb;65(2):138-44.

2. Fridkin SK, Kaufman D, Edwards JR, Shetty S, Horan T. Changing incidence of Candida bloodstream infections among NICU patients in the United States: 1995-2004. Pediatrics. 2006 May;117(5):1680-7.

**Fig. 1 (abstract A29).**
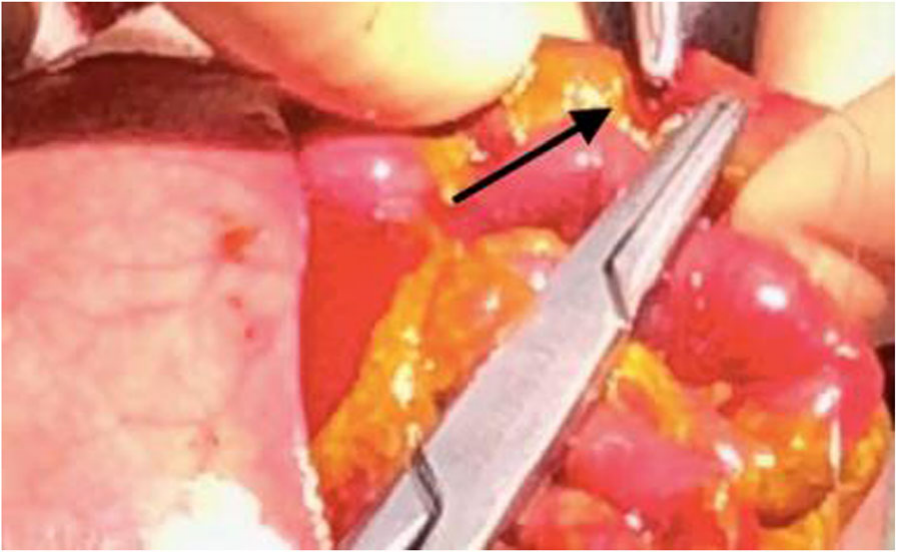
See text for description.

**Fig. 2 (abstract A29).**
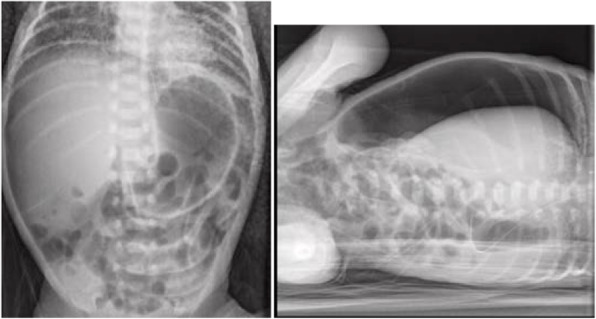
a (anteroposterior projection) and b (laterolateral projection)

## A30 Congenital neonatal ichthyosis: not such a rare disease

### Giuliana Bianchi, Ilaria D’Amico, Laura Morlacchi, Angela Bossi, Massimo Agosti

#### NICU - Woman and Child Department, F. Del Ponte Hospital, 21100 Varese, Italy.

##### **Correspondence:** Laura Morlacchi (laura.morlacchi@asst-settelaghi.it)

**Background**

Ichthyosis is an inherited heterogeneous group of disorders caused by defects in skin barrier function. [1] There are three main forms of ichthyosis [2]: common (vulgaris and X-linked); autosomal recessive congenital (ARCI): harlequin, lamellar, congenital erythroderma, bathing suit; epidermolytic hyperkeratosis (EHK). ARCI and EHK represent the uncommon forms with a prevalence of 1/100000 and 1/30000 respectively. ARCI involves a mutation in the Transglutaminase 1 (TGM1) gene. In EHK, autosomal dominant disease, mutations of genes coding for keratin proteins KRT1 and KRT10 are the most involved.

**Case reports**

C1, female infant born at 40+4 weeks of gestation by vaginal delivery after physiologic pregnancy from non-consanguineous parents. No family history of ARCI. Apgar 8, 9. Physical examination revealed cream-coloured dense platelike keratotic scale punctuated by deep red fissures, severe ectropion, eclabium, flattened nose and ear cartilage and characteristic odor of the skin (figure 1). Dermatologic treatment included daily potassium permanganate bathing, compress with physiologic solution and abundant application of emollient (paraffin) and ophthalmic gel. Analgesic therapy was conducted. Incubator humidity, initially high (90%), was progressively decreased according to a specific protocol. [3] The newborn presented gradual desquamation of the collodion membranes. At 20 days of life she developed a clinical sepsis, blood culture positive for methicillin resistant staphylococcus aureus, successfully treated with parenteral antibiotic therapy. Genetic evaluation revealed a homozygous variant c.1055C>T of TGM1 gene, not yet described in literature but included in ClinVar database as possible variation associated with lamellar ichthyosis. C2 is a male infant born at 36 weeks of gestation by vaginal delivery from a mother affected by congenital epidermolytic ichthyosis, autosomal dominant mutation c.466C>T of gene KRT10. Parents refused fetal genetic screening. Apgar 9,10. At birth the infant showed diffuse erythema and blistering with painful fissuring (figure 2a e figure 2b). Ectropion and eclabium were absent. Therapy included daily potassium permanganate bathing, abundant application of emollient (paraffin) and of topical antibiotic (fucisid acid) and analgesic treatment.

At genetic evaluation the same mutation of the mother was observed.

**Conclusions**

These clinical presentations of two different forms of congenital ichthyosis suggest that this heterogeneous disorder could be less rare than supposed. Not only are the genetics and physiology important but also the family psychological and social impact needs to be considered, alongside bacterial overlaps prevention.

Informed consent to publish has been obtained from this patient.

**References**

1. Glick JB, Craiglow BG, Choate KA, Kato H, Fleming RE, Siegfried E, Glick SA. Improved Management of Harlequin Ichthyosis With Advances in Neonatal Intensive Care. Pediatrics. 2017 Jan;139(1).

2. Peter Rout D, Nair A, Gupta A, Kumar P. Epidermolytic hyperkeratosis: clinical update. Clin Cosmet Investig Dermatol. 2019 May 8;12:333-344

3. Nguyen MA, Gelman A, Norton SA. Practical Events in the Management of a Collodion Baby. JAMA Dermatol. 2015 Sep;151(9):1031-2.

**Fig. 1 (abstract A30).**
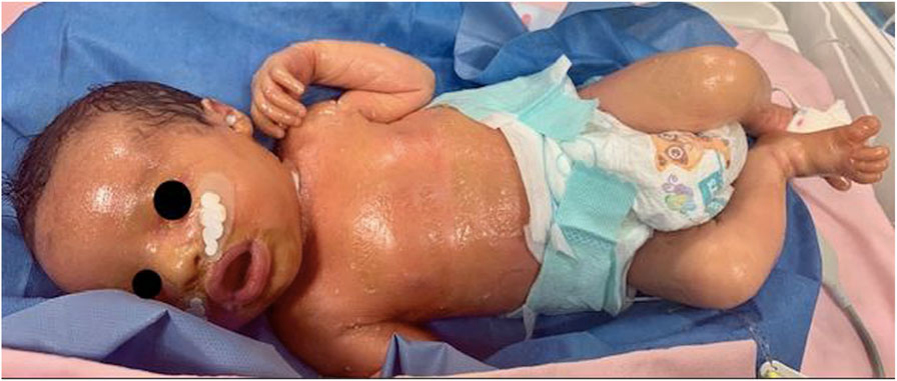
Patient C1- Lamellar ichthyosis

**Fig. 2 (abstract A30).**
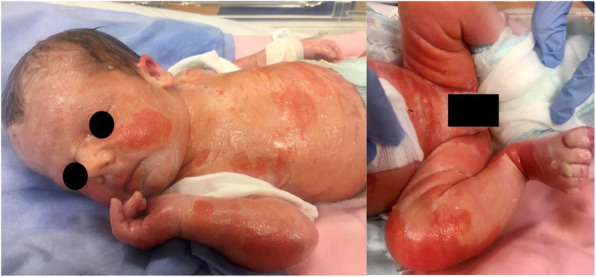
a e b. Patient C2- Epidermolytic ichthyosis

## A31 Conservative treatment of post infectious giant pneumatoceles: the right choice

### Simona Perniciaro^1^, Maria Ragazzo^1^, Francesca Paola Dessimone^1^, Giorgio Farris^2^, Mirella Mogiatti^2^, Angela Bossi^1^, Valerio Gentilino^2^, Massimo Agosti^1^

#### ^1^NICU – Woman and Child Department, F. Del Ponte Hospital, 21100 Varese, Italy; ^2^Pediatric Surgery Unit – Woman and Child Department, F. Del Ponte Hospital, 21100 Varese, Italy

##### **Correspondence:** Simona Perniciaro (simona.perniciaro@asst-settelaghi.it)

**Background**

Pneumatoceles, although rare, can occur in neonates and children as complications of severe necrotizing pneumonia or mechanical injury.[1] *Streptococcus pneumoniae* and Panton Valentine leukocidin-*Staphylococcus aureus* (PVL-SA) are the most common causative infectious agents in term newborns. [2] Giant pneumatoceles show cystic air accumulation caused by check valve mechanism and can lead to life-threatening condition with cardio-respiratory insufficiency. [3]

**Case report**

E.A. (GA 40^+3^, WT 3610 g) was full term male infant discharged in well conditions at 5^th^ day of life.

At day 15^th^ he was admitted with RSV-bronchiolitis complicated with left pneumonia and he has been treated with high flow nasal cannula and antibiotic therapy (7 days of ampicillin + sulbactam and amikacin) followed by good recovery. Blood culture was negative. At day 38^th^ he was re-admitted in NICU with fever, poor feeding, plaintive crying and groan. Despite apparently normal lung sounds, X-Ray detected lobar left pneumonia with abscess (figure 1) and chest CT with contrast showed necrotizing pneumonia with air and liquid-filled cavitations (figure 2). Blood, urine and liquor culture, pneumococcal urinary antigen and nasopharyngeal swab returned negative and tuberculosis was ruled out. During the hospitalization the baby did not need ventilatory support, but he was fed by NG-tube. Antibiotic treatment with vancomycin and ceftazidime was performed for 11 days with normalization of inflammatory index (CRP 215 mg/L->0,9 mg/L; WC 22.210/mm3->11.570/mm3). Following the clinical recovery, the antibiotic therapy was switched to oral linezolid and cefpodoxime and the baby was discharged with follow-up. After ten days, he came back again at our hospital with respiratory distress but without any infection’s signs at sepsis work-up. Chest radiography showed giant pneumatoceles with right mediastinal deviation (figure 3). Immediately CT was performed and confirmed enlarging multiple pneumatoceles in superior and inferior left lobes (biggest cyst sizes:55x40x48 mm) with severe lung atelectasis (figure 4). In agreement with the surgical team, drainage was positioned in the major air-filled cyst keeping low pressure suction with an initial partial improvement and subsequently closed and removed after few days. Antibiotic treatment was continued with clindamycin for three weeks. According to colleagues of surgical team and considering the baby’s stability, the “wait and see” strategy was preferred. Three weeks after admission the baby was healthy, fully oral fed and chest X-Ray indicated a partial reduction of pneumatoceles and a central mediastinum (figure 5).

**Conclusion**

Most pneumatoceles resolve spontaneously within one years and may be treated conservatively [4] with prolonged antibiotic therapy. Surgical treatment could be reserved for critical baby with complicated pneumatoceles characterized by more than 50% involvement of the hemithorax, frequent chest infections or bronchopleural fistula.

Informed consent to publish has been obtained from this patient.

**References**

1. Spencer DA, Thomas MF. Necrotizing pneumonia in children. Paediatric Respiratory Reviews (2013), 10.1016/j.prrv.2013.10.001

2. Liu C, Bayer A, et al Clinical practice guidelines by Infectious Diseases Society of America for the Treatment of Methicillin-Resistant *Staphylococcus aureus* Infections in Adults and Children. Clinical Infectious Diseases. 2011:52 (1 February)

3. Park TH, Kim JK. Nonsurgical Management of an Enlarging Pneumatocele by Fibrin Sealant Injection via Pigtail catheter. Pediatric Pneumology. 2016;51: E5-E7

4. Gross I. Gordon O, et al. Giant lung cysts following necrotizing pneumonia: Resolution with conservative treatment. Pediatric Pulmonology. 2019;54: 901-906

**Fig. 1 (abstract A31).**
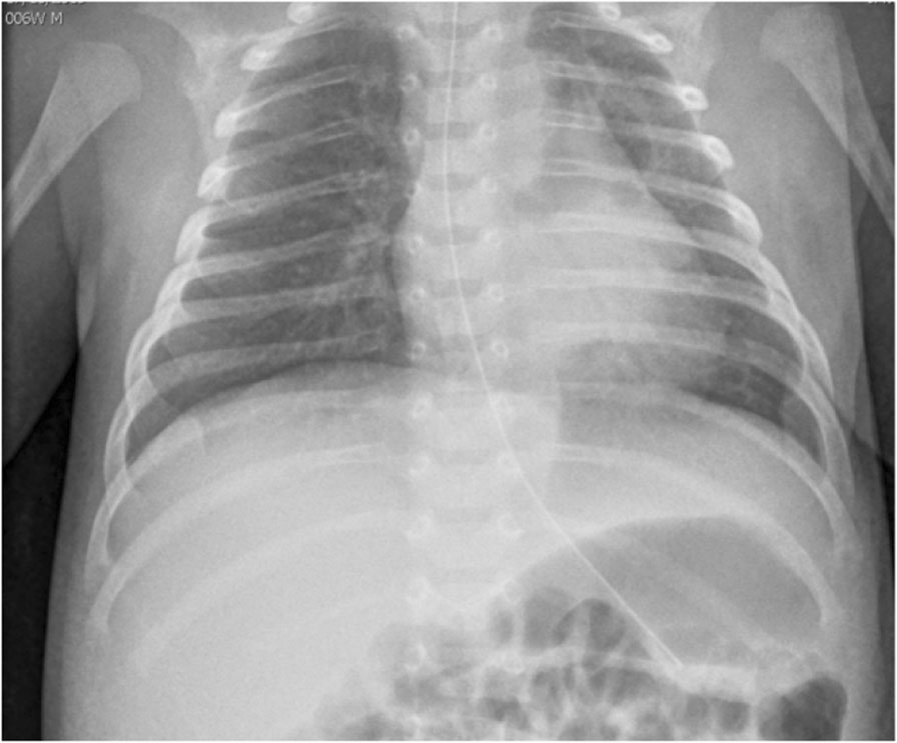
See text for description

**Fig. 2 (abstract A31).**
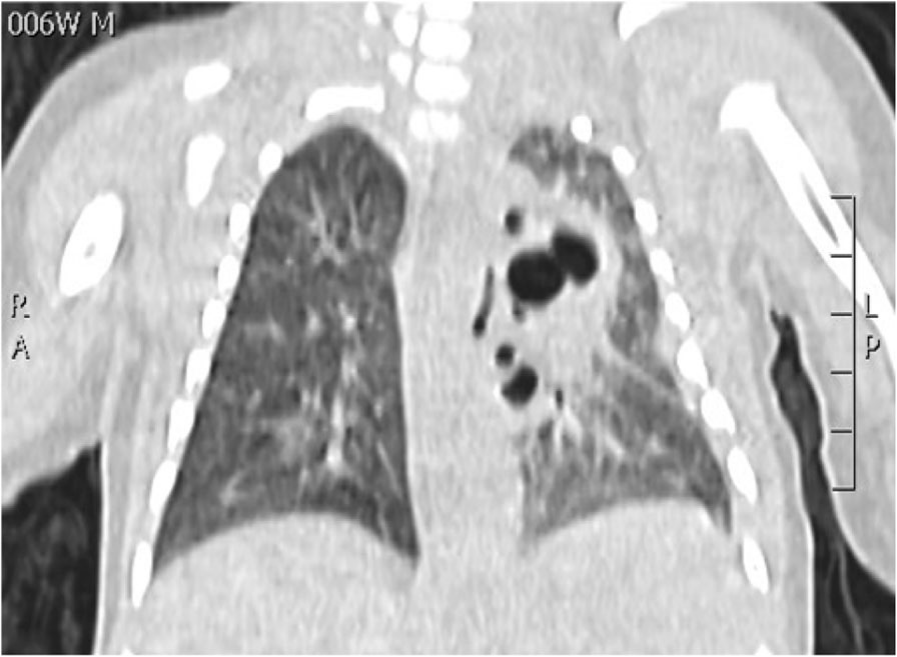
See text for description

**Fig. 3 (abstract A31).**
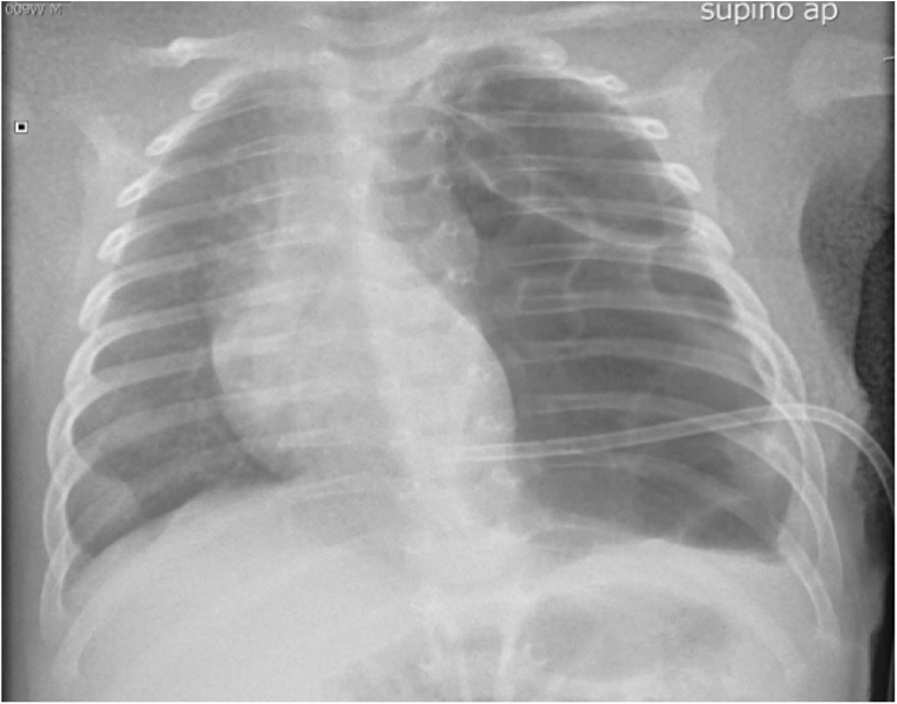
See text for description

**Fig. 4 (abstract A31).**
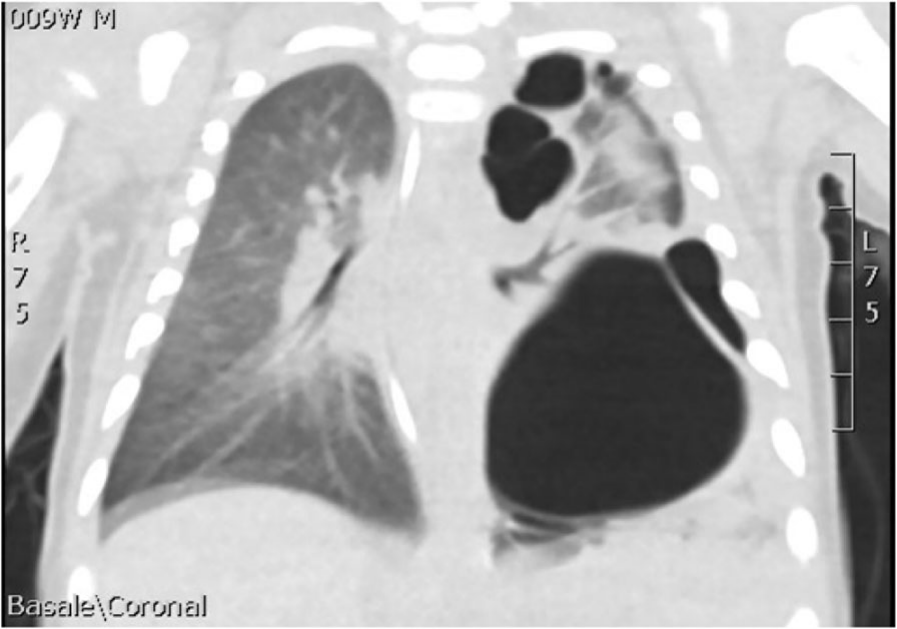
See text for description

**Fig. 5 (abstract A31).**
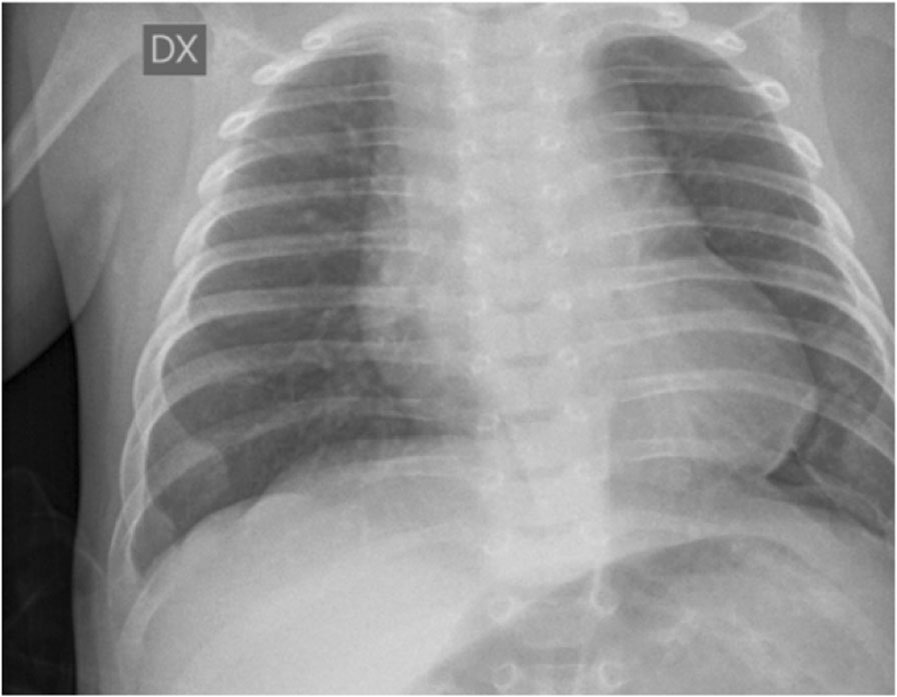
See text for description

## O6 Incidence of medication errors in a Neonatal Intensive Care Unit

### Cecilia Novara^1,2^, Elena Ciarmoli^1^, Emanuela Scalmani^1^, Gian Luigi Marseglia^2^, Paolo Ernesto Villani^1^, Maria Pierro^1^

#### ^1^NICU, Mother’s and baby’s health department, Fondazione Poliambulanza Istituto Ospedaliero, Brescia Italy; ^2^Department of Paediatrics, IRCCS Policlinico San Matteo, Pavia, Italy

##### **Correspondence:** Maria Pierro (maria.pierro@poliambulanza.it)

**Background**

The issue of pharmacological errors is of considerable relevance in the paediatric field. However, the data concerning the real incidence of medical errors (MEs) in the paediatric population are sparse and inconsistent [1-4]. Undoubtedly, newborn infants are at greatest risk of MEs and preventable adverse events. A deeper knowledge on the epidemiology of ME in neonates may help their prevention.

The aim of the study is to prospectively record and analyse the incidence of MEs in our NICU.

**Material and methods**

From October to December 2019, we prospectively collected all the MEs. MEs were identified by (i) spontaneous reporting (ii) daily audit and (iii) daily chart revision.

In our NICU the therapeutic process is currently managed through a paper-order entry charting.

**Results**

During the study period we detected 111 ME out of 1914 drug prescriptions (5,55%). The medications that were most frequently associated with errors were: anti-reflux, heparin, endovenous solutions, electrolyte corrections, diuretics and inotropes (37% 29%, 26%, 23%, 18% respectively out of each specific drug total prescriptions,) followed by steroids, antibiotics, opioids, caffein and vitamins (11%, 10%, 8,8%, 4,8% respectively). Types of errors were classified as follows: wrong drug (27%), missing nurse check of therapy administration (22%), wrong dose (15%), missing prescription (10%), missing drug interruption (8%), missing administration (5%), wrong frequency (5%), wrong route of administration (1%). With regards to the phase of the therapeutic process, 47% were prescription errors and 53% administration errors. We did not register any preparation error, most likely because those are more difficult to detect. There was no association with the route of administration, number of prescribed drugs per patient and years of experience of the involved personnel. On the contrary, ME were strictly related to the patients/nurses ratio: 6,1% vs 9,1 vs 17,1 for a 2:1, 3:1, and 4:1 ratio respectively. More than 50% of the MEs were intercepted by the medical personnel before getting to the patient. We did not detect any adverse event related to the MEs.

**Conclusions**

Our data are consistent with those of the literature. Although we did not observe any preventable adverse event, efforts should be made to reduce medication errors and near-misses, in the attempt of avoiding potential neonatal complications. Among several strategies, an adequate patients/nurses ratio is critical in order to ensure a safe stay in the NICU.

**References**

1. Kaushal R, Bates DW, Landrigan C, McKenna KJ, Clapp MD, Federico F, et al. Medication errors and adverse drug events in pediatric inpatients. Jama. 2001;285(16):2114-20.

2. Marino BL, Reinhardt K, Eichelberger WJ, Steingard R. Prevalence of errors in a pediatric hospital medication system: implications for error proofing. Outcomes management for nursing practice. 2000;4(3):129-35.

3. Priyadharsini R, Surendiran A, Adithan C, Sreenivasan S, Sahoo FK. A study of adverse drug reactions in pediatric patients. Journal of pharmacology & pharmacotherapeutics. 2011;2(4):277-80.

4. Ross LM, Wallace J, Paton JY. Medication errors in a paediatric teaching hospital in the UK: five years operational experience. Archives of disease in childhood. 2000;83(6):492-7.

## O7 Right ventricular dominance and interrupted inferior vena cava as an isolated prenatal finding: the importance of a fetal and neonatal integrated echocardiographic management

### Elena Portinaro^1,3^, Stefano Fiocchi^1^, Cristina Della Valle^2^, Laura Guzzetti^2^, Daniele Merazzi^1^

#### ^1^UOC di Neonatologia e Terapia Intensiva Neonatale, Ospedale Valduce, Como ) (Italy); ^2^UOC di Ostetricia e Ginecologia, Ospedale Valduce, Como ) (Italy); ^3^Università Tor Vergata, Roma ) (Italy)

##### **Correspondence:** Elena Portinaro (elena.portinaro@gmail.com)

**Background**

Fetal venous drainage is made up by two horns: the right and the left one. Each one is formed by umbilical, cardinal and vitelline veins and they are initially symmetric. The transition to an asymmetrical system typical of adults is linked to a remodeling caused by the left-right venous shunt [1]. Most of these veins undergo resorptive degeneration and finally we can distinguish the superior and the inferior cava veins. Interrupted inferior cava vein (ICV) with azygous continuation is the result of connection failure between the right subcardinal vein and the right vitelline vein [2,3]. Usually this is an excellent prenatal marker of heterotaxy and polysplenia syndrome [3,4] linked to this condition in 85% cases and rarely to asplenia syndrome (Van Praagh collection). Isolated interrupted ICV with azygous continuation is a rare condition prenatally detectable with a benign neonatal outcome.

**Case Report**

A quartigravida 21 y.o. woman was followed-up by serial US scans since 32 weeks of gestational age of pregnancy for suspected fetal aortic coartaction. The scans revealed right dominant heart [5], tricuspidal regurgitation, azygous continuation in absence of ICV in *situs solitus*. A 2010 g (SGA, < 3^rd^ centile) female infant was delivered with Apgar 6/9. At birth she was supported by n-CPAP ventilation with maximum FiO_2_ of 0.7 for 3 days. Differential pre-post ductal pulse oximetry was > 10 points for at least 48 hours. Neonatal echocardiography: enlargement of right ventricle with poor function and large PDA (4 mm) with exclusive right to left shunt, absence of ICV and dilated superior cava vein with azygous continuation.

Neonatal management was based on frequent functional echocardiographic scans to monitor the ductal evolution and to assess right ventricular function [6,7,8]. Day III-IV: inversion of ductal shunt with systemic hypotension and poor diastolic function requiring inotrope support with dopamine till VI day of life. Day V-VII: ductal restriction with exclusive left-to-right shunt and improvement of right cardiac function; regular aortic arch. Clinical condition improved progressively and weaning of n-cpap support and oxygen supplementation were possible.

**Conclusion**

Interruption of the ICV with azygous continuation represents the most common abnormality involving fetal vein system [3] also as an isolated finding demonstrable by echo with “double vessel sign” [4]. An hypoxic condition due to fetal growth restriction could explain the slower than expected maturation of pulmonary vascular resistance in the late gestation and the right ventricular impairment [9,10]. Echo daily monitoring allowed us to tailor the neonatal management following step-by-step the fetal-neonatal transition.

Consent to publish has been obtained from the parents

**References**

1. Moore KL, Persuad TVN, Torchia MG. The developing human. Clinical oriented embryology. 9^th^ edn. Elsevier, 2013, 333-7

2. Sadler TW. Cardiovascular system. In Langman’s Medical Embriology, 6^th^ edn. Baltimore: Wiliams & Wilkins, 1990, 217-20

3. Celentano C, Malinger G, Rotmensch S, Gerboni S, Wolman Y, Glezerman M. Prenatal diagnosis of interrupted inferior vena cava as an isolated finding a benign vascular malformation. Ultrasound Obstet Gynecol 1999, 14:215-218

4. Sheley RC, Nyberg DA, Kapur R. Azygous continuation of the interrupted inferior vena cava: a clue to prenatal diagnosis of the cardiovascular syndromes. J Ultrasound Med 1995, 14:381-7

5. Feng J, Zhu M, Liang H, Li Q. Prenatal diagnosis of right dominant heart in fetuses: a tertiary center experience over a 7-year period. Chinese Med J 2017, vol 130, 574-580

6. J. E.B. Cabral, J. Belik: ”Persistent pulmonary hypertension of the newborn: recent advances in pathophysiology and treatment”, review, J Pediatr (Rio J), 2013

7. R. H. Steinhorn: “Neonatal pulmonary hypertension”, Pediatr Crit Care Med, 2010

8. S. Stewart, D. Rassl: “Advances in the understanding and classification of pulmonary hypertension”, Review, Histopathology, 2009

9. Cruz-Lemini M, Crispi F, Valenzuela-Alcaraz B, Figueras F, Sitges M, Bijnens B et Al. Fetal cardiovascular remodeling persists at 6 months in infant with intrauterine growth restriction. Ultrasound Obstet Gynecol 2016, 48: 349-56

10. Akazawa Y, Hachiya A, Yamazaki S, Kawasaki Y, Nakamura C, Takeuchi Y et Al. Cardiovascular remodeling and dysfunction across a range of growth restriction severity in small for gestational age infants – Implications for fetal programming. Circ J 2016; 80: 2212-20

## A32 Safety and efficacy of a novel scheme of thrombolysis in neonates

### Alessandra Rossi^1, 2^, Elena Ciarmoli^1^, Emanuela Scalmani^1^, Paolo Villani^1^, Gian Luigi Marseglia^2^, Mariella Magarotto^3^, Maria Pierro^1^

#### ^1^Department of Mother's and Child's Health, Fondazione Poliambulanza Istituto Ospedaliero, Brescia, Italy; ^2^Department of Pediatrics, Fondazione IRCCS Policlinico San Matteo, University of Pavia, Pavia, Italy; ^3^Neonatal Intensive Care Unit, Pediatric University Hospital, Padua, Italy

##### **Correspondence:** Maria Pierro (maria.pierro@poliambulanza.it)

**Background**

Severe thrombotic events are a rare complication in newborn infants. Currently, there is no consensus regarding the optimal treatment scheme for thrombosis in neonates. One well-known therapeutic approach is urokinase up to 3 days. However, inefficacy or thrombus rebound, may have long-term detrimental consequences on organ development. We present a case of an ECMO related life-threatening massive thrombosis. The infant, was successfully treated with a longer scheme of urokinase, guided by vascular ultrasound and coagulation results.

**Case Report**

A male neonate was born at term by emergency C-section because of fetal bradycardia and meconium-stained amniotic fluid. Extensive neonatal resuscitation was required at birth. The baby developed a severe meconium aspiration syndrome, requiring ECMO from 28 hours of life for 4 days, with optimal response. At 1 week of life, a right internal jugular vein thrombus was detected on ultrasound. Therefore, enoxaparin was started. On the 18th day of life, the right lower limb became tender, warm and markedly swollen. A vascular ultrasound showed an occlusive thrombus in the right common femoral vein extending into the external iliac vein. Therefore, unfractionated heparin was increased. On the 19th day of life, the respiratory status dramatically deteriorated and acute renal insufficiency developed. C-reactive protein was highly increased, while procalcitonin was negative, suggesting a non-infective pro-inflammatory status. A contrast CT-scan confirmed the hypothesis of a massive thrombosis of the superior and inferior vena cava affecting renal, iliac and femoral veins. Therapy with urokinase was started, leading to a rapid clinical improvement. During the thrombolytic treatment the baby was monitored by daily blood work and ultrasound. The treatment was prolonged until evidence of thrombolysis (increased D-dimer plus steady recanalization of thrombi on ultrasound) was present. After 21 days of treatment, d-dimer drastically decreased, while vein recanalization had been stable for 48 hours. Since thrombolysis ceased, thrombolytic treatment was stopped and anticoagulation treatment was resumed. The baby was discharged at 3 months of life in overall good health, spontaneously breathing in room air, with a normal neurological exam and no evidence of thrombi rebound.

**Conclusions**

In our report urokinase was safe and effective up to 21 days. Vascular ultrasound and coagulation results seemed to be consistent among each other. Our case paves the way for trials aiming to investigate the effectiveness of a targeted anti-thrombotic therapy, based on evidence of thrombolysis.

Informed consent to publish has been obtained from this patient.

## A33 Unusual stridor and apnoea in newborn: a thymus where it shouldn’t be

### Rosario Ippolito^1^, Enrico Tondina^1^, Chiara Gertosio^1^, Mariasole Magistrali^1^, Maria Sole Prevedoni^2^, Lucia Schena^1^, Claudia Viganò^3^, Alberto Chiara^3^

#### ^1^ Neonatal Intensive Care Unit, IRCCS San Matteo Hospital Foundation, University of Pavia, Italy; ^**2**^ Pediatric Radiology, Dep. of Diagnostic Medicine, IRCCS San Matteo Hospital Foundation, Pavia, Italy; ^**3**^ Neonatal Intensive Care Unit, IRCCS San Matteo Hospital Foundation, Pavia, Italy.

##### **Correspondence*:*** Enrico Tondina (e.tondina@gmail.com)

Ippolito R. and Tondina E. contributed equally to the writing of the work.

**Background:**

Aberrant cervical thymus is a rare clinical entity that results from an incomplete migration of the thymic tissue during the last phases of human embryogenesis. By result, thymic tissue can be found in any location along the usual pathway of thymic descent, from pharynx to mediastinum. Usually it manifests itself as a cystic or solid mass, sideways to the sternocleidomastoid muscle, free of infiltration or contiguity with the greater vascular axis of the neck. [1, 2]

**Case report**

V.A., a male newborn, was born at full-term by caesarean section, after an uneventful pregnancy. Twenty days after birth, he was evaluated in our intensive care nursery for apnoea and face cyanosis episodes, occurred for few seconds during sleeping, with recovery after stimulation. Regurgitation episodes and asymmetry of the palpebral fissure have been reported. Clinically he had left periorbital edema and strabismus, as well as inspiratory stridor during crying.

He was diagnosed gastro-esophageal reflux disease (GERD) by first digestive tract radiography and he started therapy with proton pump inhibitor (PPI) with benefit. Ophthalmic and neurological evaluation were performed for eye signs with clinical confirmation of asymmetry of the ocular globes with lagophthalmos on the left. Orbital ultrasonography (US) wasn’t diagnostic, so a ocular orbits magnetic resonance imaging (MRI) was performed. MRI showed a lateral-cervical space-occupying lesion, with dimensions of 20x18x39 mm, clear edges, compatible with supernumerary/ectopic latero-cervical thymus. Therefore neck ultrasonography (US) was also performed. This showed a lateral-cervical space-occupying lesion with ecogenicity similar to the thymus’ one in its usual place. Blood determination of tumoral markers was negative.

It was performed surgically and otolaryngology assessment with laryngoscopy performance that excluded surgical indication and suggested a close follow-up.

**Conclusions**

Neck masses in the neonatal period often represent a real clinical challenge, although more than 80% of them have a benign histology. Most of times these masses are asymptomatic, but in some cases they can dislocate the main structures of the neck causing stridor and recurrent episodes of upper airway obstruction (and apnoea). For this reason, in addition to a careful clinical evaluation and a precise diagnostic radiological framework, it’s undoubtedly important not understimate all those cases laterocervical masses in the newborn that present a progressive course, for which a surgical evaluation is often required.

Informed consent to publish has been obtained from this patient.

**References:**

1. Paraboschi I, Fati F, Rizzo F, Sacco O, Stagnaro N, Mattioni G, Simonini A, Mazzei O, Torre M. Ectopic Thymus: an unusual case of subglottic mass. Annals of Otology, Rhinology & Laryngology. 2019*, Vol.128 (12) 1182-1188.*

2. Purcelli PL, Marquez Garcia J, Zawawi F, Propst EJ, Pansini BC, Blaser SI, Wolter NE. Ectopic cervical thymus in children: clinical and radiographic features. Laryngoscope, 00:1-6, 2019.

## A34 Kleihauer-Betke: clinical usefulness and reliability of a simple test

### Elisa Tota^1^, Roberto Romano^1^, Sara El Oksha^2^, Carlo Maria Brambilla di Civesio^2^, Gianandrea Rettani^2^, Diana Ghisleni^2^, Giuseppe Banderali^2^

#### ^1^Department of Health Sciences San Paolo Hospital University of Milan ((Italy); ^2^Pediatric Unit San Paolo Hospital ASST Santi Paolo Carlo Milano (Italy)

##### **Correspondence:** Elisa Tota (elisa.tota@unimi.it)

**Background**

Feto-maternal hemorrhage (FMH) is a known cause of anemia in the fetus and in the newborn. It refers to the entry of fetal blood into the maternal circulation before or during delivery. Very small amount of fetal red cells are normally detectable in all pregnancies. FMH has a very wide spectrum of clinical variation depending on hemorrhage’s severity.

**Case report**

We hereby report 2 cases of FMH with very different outcomes. M.M., female born at term (39+5 eg weeks) by spontaneous delivery after normal pregnancy, admitted in the Neonatal Pathology Unit for a very early and unexpected condition of anemia (umbelical cord blood gas analysis [BGA] with low hemoglobin and hematocrit levels, 8.7 g/dl and 26% respectively). APGAR score 1’ 10, 5 ’10, birth weight 3350g. Cell blood count (CBC) confirmed anemia (Hb 9.6 g/dl, Ht 32.3%) with associated elevated reticulocytes count (8.40%). A blood smear was performed and turned out negative for red cells membrane defects. At 4 hours oxygen supply for respiratory distress and low SpO2 (87-90%) was needed; no tachicardia. Abdomen US showed perihepatic and spleen effusion; no evidence of brain bleeding. Being a symptomatic anemia, red blood cell transfusion was performed via umbilical venous catheter. Direct Coombs test was negative and TORCH infections were excluded. Kleihauer-Betke test was thus performed, showing 4% and making the diagnosis of a transfusion from fetus to mother. Baby’s clinical condition fastly improved and she was discharged briefly after. N.F., male born at term (40+1 eg weeks) after normal pregnancy by urgent Caesarean Section for fetal bradycardia, admitted in neonatal intensive care unit for a condition of asphyxia and severe anemia at birth. APGAR score 1’ 0, 5’ 0, 10’ 3. Umbelical cord BGA showed severe acidosis (pH 6.74) and hemoglobin was 2.6 mg/dl. He died few days after. During pregnancy there was no evidence of placental abruption. Kleihauer-Betke test was performed and came out strongly positive (9%) thus diagnostic.

**Conclusions**

FMH can be a subtle cause of anemia in the newborn and strongly different outcomes can be expected based on the loss of fetal blood, which is directly correlated to the percentage of Kleihauer-Betke test, as suggested by our reported cases. Since potential life threatening conditions can be related to FMH, we advise to always consider Kleihauer-Betke test in the differential diagnosis of feto-neonatal anemia especially if no other causes of anemia are clearly detected.

Informed consent to publish has been obtained from parents of both patient.

## O8 3 cases of cow's milk protein intolerance in the first week of life

### Serena Ossola, Valeria Trivellin, Maria Ragazzo, Angela Bossi, Massimo Agosti

#### NICU - Woman and Child Department, F. Del Ponte Hospital, Varese, Italy.

##### **Correspondence:** Valeria Trivellin (valeria.trivellin@asst-settelaghi.it)

**Background**

The incidence of cow’s milk protein (CMP) allergy in the first year of life is 2-7%. Allergy or intolerance to CMP in neonatal age are widely described in the literature [1, 2], but only few cases of prenatal sensitization and demonstrated symptoms in the first week of life are reported [3]. We describe 3 very early onset cases occurred at our department in the last year.

**Case reports**

C1 was born at 35 weeks gestational age (GA) by vaginal delivery because of spontaneous labor following physiological pregnancy. Good adaptation to extrauterine life, birth weight was appropriate for GA. At 15 minutes of life emission of plenty bright red blood in meconium, in good clinical conditions. C2 and C3 were born at term after physiological pregnancy, presenting normal Apgar score and birth weight. C2 was discharged from hospital in 3^rd^ day of life in physiological weight loss, exclusively breastfed. In the 4^th^ day of life appearance of plenty bright red blood in the stool in good clinical conditions. C3 was fed with exclusive maternal breast milk in the first 2 days of life. In his 3^rd^ day of life, in good clinical conditions, mucus and plenty bright red blood in the stool were observed. Despite persistently normal physical examination, all 3 newborns have been admitted in Neonatal Intensive Care Unit with exclusive parenteral nutrition for 3 days. The only abnormal investigation observed in each case was mild leucocytosis with eosinophilia at complete blood count (maximum eosinophils 3400/mm3 in C1, 2400/mm3 in C2, 1800/mm3 in C3). The remaining blood tests (inflammation indexes, coagulation, liver and kidney function), blood and stool cultures and the research for fecal viral antigens were negative. Abdominal ultrasound was normal in C1 and C3, showed mild thickening of colon and rectal mucosa in C2; abdominal X-ray in all cases and contrast X-ray in C1 were normal. An attempt to reintroduce maternal colostrum and preterm formula milk in C1 caused episodes of vomiting and reappearance of bloody feces, which finally stopped 2 days after introduction of elementary diet. The mother of C2 was put on a CMP free diet and 3 days after reintroduction of breastfeeding no more blood was observed in the stools. In C3, because of insufficient breast milk, integrations with extensively hydrolysed formula milk were administered. In the following 3 days the feces normalized. At 5 months of life C1’s blood count normalized, skin prick tests for CMP were negative and oral provocation test was performed, with prompt beginning of projectile vomiting. CMP intolerance was confirmed, CMP free diet continued and at 1 year of life C1 is in good clinical conditions. At 2 months of life C2’s eosinophils were normal, blood specific IgE and skin prick tests versus CMP were negative, oral provocation test with formulated milk was negative and normal maternal diet could be restarted.

C3 control blood count and oral provocation test are scheduled for the 3^rd^ month of life.

**Conclusions**

The early onset of symptoms in our 3 patients supports the hypothesis, rarely described in the literature, that allergic immunization can occur in utero.

Informed consent to publish has been obtained from parents of all patients.

**References:**

1. Different presentations of cow’s milk protein allergy during neonatal period Selma Aktas et al. The Turkish Journal of Pediatrics 2017; 59: 322-328

2. Manifestation of cow’s milk protein intolerance in preterm infants. Cordova et al. JPGN 2016; 62: 140-144.

3. Neonatal Eosinophilic Gastroenteritis: Possible in Utero Sensitization to Cow’s Milk Protein. Humam S. Alabsi et al. Neonatal Netw. 2013 Sep-Oct;32(5): 316-22

